# Reducing brassinosteroid signalling enhances grain yield in semi-dwarf wheat

**DOI:** 10.1038/s41586-023-06023-6

**Published:** 2023-04-26

**Authors:** Long Song, Jie Liu, Beilu Cao, Bin Liu, Xiaoping Zhang, Zhaoyan Chen, Chaoqun Dong, Xiangqing Liu, Zhaoheng Zhang, Wenxi Wang, Lingling Chai, Jing Liu, Jun Zhu, Shubin Cui, Fei He, Huiru Peng, Zhaorong Hu, Zhenqi Su, Weilong Guo, Mingming Xin, Yingyin Yao, Yong Yan, Yinming Song, Guihua Bai, Qixin Sun, Zhongfu Ni

**Affiliations:** 1 Frontiers Science Center for Molecular Design Breeding, China Agricultural University, Beijing, China; 2 National Observation and Research Station of Agriculture Green Development (Quzhou, Hebei), China Agricultural University, Beijing, China; 3 USDA-ARS, Hard Winter Wheat Genetics Research Unit, Manhattan, KS USA

**Keywords:** Plant breeding, Brassinosteroid, Plant genetics, Agriculture, Sustainability

## Abstract

Modern green revolution varieties of wheat (*Triticum aestivum* L.) confer semi-dwarf and lodging-resistant plant architecture owing to the *Reduced height-B1b* (*Rht-B1b*) and *Rht-D1b* alleles^1^. However, both *Rht-B1b* and *Rht-D1b* are gain-of-function mutant alleles encoding gibberellin signalling repressors that stably repress plant growth and negatively affect nitrogen-use efficiency and grain filling^2–5^. Therefore, the green revolution varieties of wheat harbouring *Rht-B1b* or *Rht-D1b* usually produce smaller grain and require higher nitrogen fertilizer inputs to maintain their grain yields. Here we describe a strategy to design semi-dwarf wheat varieties without the need for *Rht-B1b* or *Rht-D1b* alleles. We discovered that absence of *Rht-B1* and *ZnF-B* (encoding a RING-type E3 ligase) through a natural deletion of a haploblock of about 500 kilobases shaped semi-dwarf plants with more compact plant architecture and substantially improved grain yield (up to 15.2%) in field trials. Further genetic analysis confirmed that the deletion of *ZnF-B* induced the semi-dwarf trait in the absence of the *Rht-B1b* and *Rht-D1b* alleles through attenuating brassinosteroid (BR) perception. ZnF acts as a BR signalling activator to facilitate proteasomal destruction of the BR signalling repressor BRI1 kinase inhibitor 1 (TaBKI1), and loss of *ZnF* stabilizes TaBKI1 to block BR signalling transduction. Our findings not only identified a pivotal BR signalling modulator but also provided a creative strategy to design high-yield semi-dwarf wheat varieties by manipulating the BR signal pathway to sustain wheat production.

## Abstract (web summary)

A strategy that depends on attenuated brassinosteroid signalling is described for the design of semi-dwarf wheat varieties with improved grain yield compared with that of green revolution varieties.

## Main

The green revolution in the 1960s has markedly increased cereal crop yield through widespread cultivation of semi-dwarf and lodging-resistant varieties^1,6^. The beneficial semi-dwarf plant architecture of these green revolution varieties (GRVs) is mainly conferred by the introduction of either of the *Reduced height-1* (*Rht-1*) alleles (*Rht-B1b* or *Rht-D1b*) that derived from a gain-of-function mutation of *Rht-B1a* in the B genome or *Rht-D1a* in the D genome of wheat (*Triticum aestivum* L., 2*n* = 6*x* = 42, AABBDD genome), and a recessive mutant *semi-dwarf1* (*sd1*) in rice (*Oryza sativa* L., 2*n* = 2*x* = 24). The *Rht-B1b*, *Rht-D1b* and *sd1* alleles lead to high levels of accumulation of DELLA proteins that repress gibberellin (GA) signalling and further attenuate GA-promoted plant growth to shape semi-dwarfism^1,6^. However, these green revolution alleles also reduce nitrogen (N)-use efficiency (NUE) and carbon fixation, resulting in decreased biomass, spike size and grain weight in the GRVs^3–6^. Therefore, the GRVs require extremely high N fertilizer inputs to maintain their high yields, but high N input is detrimental to both environments and agriculture sustainability^7^. Identifying new genetic sources that produce desirable semi-dwarf plant architecture with improved NUE without plant growth and grain yield penalty is an urgent goal for continuous improvement of yields of cereal crops in the limited arable lands to feed a growing world population.

Previous studies in rice have established essential roles of the N-regulated plant-specific transcription factor GROWTH-REGULATING FACTOR 4 (GRF4) together with its coactivator GRF-INTERACTING FACTOR1 (GIF1) in activating multiple N-metabolism genes. DELLA proteins inhibit the GRF4–GIF1 activity^2,8^; however, increasing the abundance of GRF4 can repress DELLA activity to boost NUE and increase biomass and final grain yields in rice and wheat GRVs^2,9,10^. A recent study revealed that an N-induced APETALA2-domain-containing NITROGEN-MEDIATED TILLER GROWTH RESPONSE 5 (NGR5) is a key regulator for genome-wide transcriptional reprogramming in response to N fertilization, and increased *NGR5* expression in rice enhanced NUE and grain yield^4^. These studies suggest feasibility to design improved GRVs in cereal crops using the available green revolution genes.

BR has diverse roles in regulating important agronomic traits including plant architecture, spike and panicle morphology, and grain size and shape in cereal crops^10–13^. BR-deficient crops usually exhibit a dwarf and compact plant stature that is beneficial to lodging resistance and high-density planting^13–15^. Here we report a strategy to breed new wheat GRVs with more compact semi-dwarf plant architecture, improved NUE and enhanced grain yields using a rare, natural deletion of a haploblock, designated as *r-e-z*. The deleted haploblock includes *Rht-B1* and its two neighbouring genes, *EamA-B* encoding an EamA-like transporter family protein and *ZnF-B* encoding a zinc-finger RING-type E3 ligase. Our genetic and molecular data support a fundamental role of *ZnF-B* deletion in shaping the green revolution trait mainly through partial attenuation of BR signalling in the absence of both *Rht-B1b* and *Rht-D1b*.

## Identification of the rare haploblock deletion

**Fig. 1:**
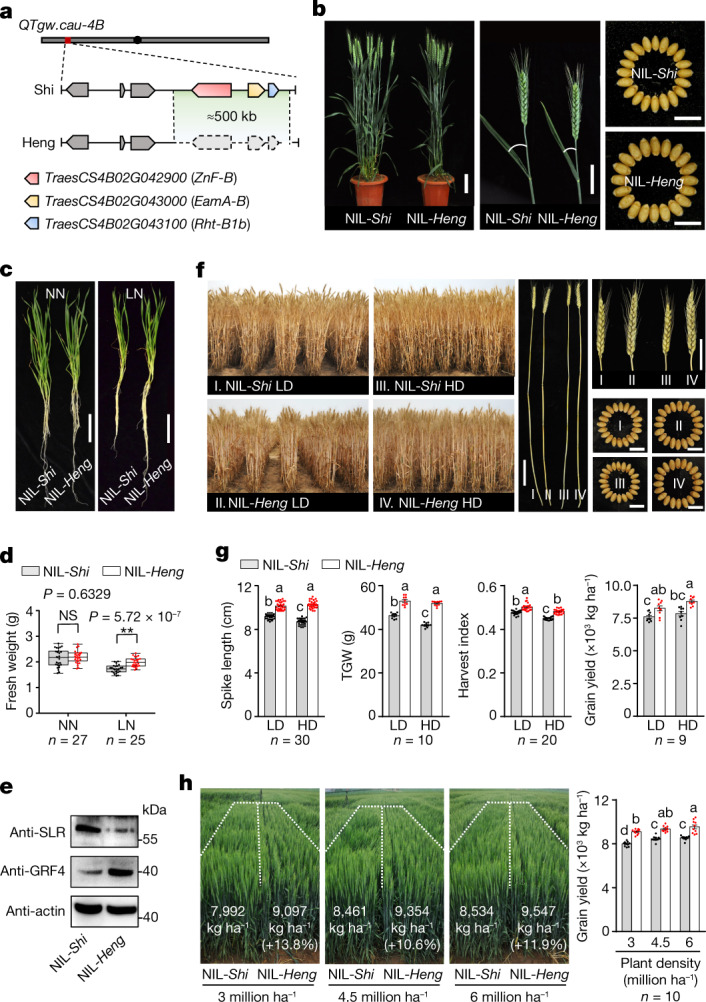
The *r-e-z* haploblock deletion improves green revolution plant architecture and grain yield in wheat. **a**, Schematic representation of chromosome 4B to show the locations (red bar) of *QTgw.cau-4B* and the *r-e-z* haploblock deletion in Shi and Heng. The *r-e-z* haploblock deleted in Heng is outlined with dashed lines. **b**, Comparison of plant height, spikes and grain sizes between NIL-*Shi* and NIL-*Heng*. **c**,**d**, Seedling plant growth performance of the two NILs under normal nitrogen (NN; 2.5 mM KNO_3_) and low-nitrogen (LN; 0.5 mM KNO_3_) conditions. The horizontal bars of the boxes represent minima, 25th percentiles, medians, 75th percentiles and maxima (**P < 0.01; NS, not significant; two-tailed Student’s *t*-test). **e**, Comparison of DELLA and GRF4 protein content between two NILs. Actin served as a loading control. The experiment was repeated independently three times with similar results. **f**,**g**, Comparison of whole plants, spikes and grains between the two NILs grown under low density (LD) with 0.3-m row space and high density (HD) with 0.15-m row space. **h**, Comparison of plant phenotypes and final yields between the NILs planted at three planting densities in a standard field. Left panel shows field plots of two NILs under three different planting densities. The field plots are outlined by dashed lines; right panel shows the grain yields of two NILs under three planting densities. In **g**,**h**, Different letters indicate significant differences (*P* < 0.05, one-way analysis of variance (ANOVA), Tukey’s honestly significant difference (HSD) test; data are mean ± s.e.m.). In **d**,**g**,**h**, *n* represents numbers of biologically independent samples. Scale bars (**b**,**c**,**f**), 10 cm for plants, 5 cm for spikes and 1 cm for grains. Source data

**Extended Data Fig. 1:**
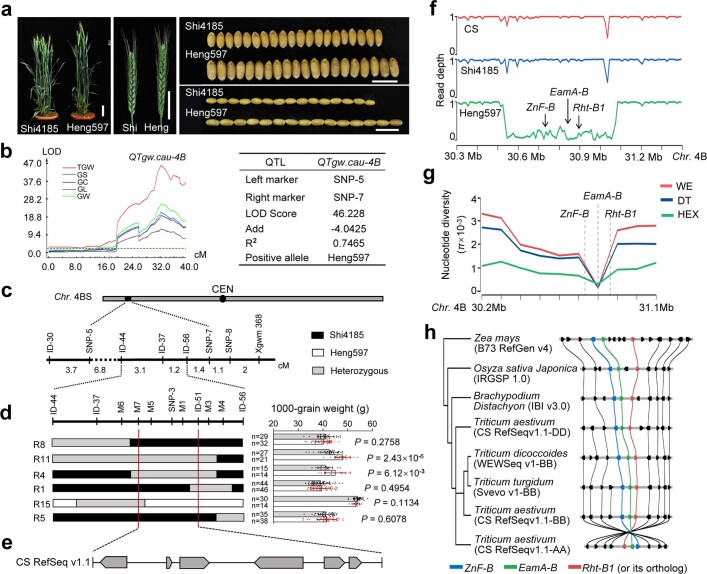
Mapping of *QTgw.cau-4B* and nucleotide diversities of the genomic region spanning *ZnF*, *EamA-B*, and *Rht-B1*. **a**, Comparison of whole plants, spikes, and grain traits between the two parents, Shi and Heng. **b**, Significant quantitative trait locus (QTL) *QTgw.cau-4B* for grain traits mapped on chromosome 4B using (Shi × Heng) segregating population. The Y- and X-axes show the LOD value and genetic positions (cM) of markers, respectively. ‘Add’ and ‘R^2^’ represent additive effects and phenotypic variation explained by the QTL, respectively. **c**, *QTgw.cau-4B* mapped in the short arm of chromosome 4B and markers mapped nearby or within the QTL region. CEN, centromere. **d**, Selected six representative recombinants between markers M7 and ID-51 (red lines) flanking *QTgw.cau-4B* and their 1000-grain weight (TGW) data (right). Data are mean ± s.d. (*n* = numbers of biologically independent samples). *P-*values were calculated by a two-tailed Student’s *t*-test. **e**, Within the delimited *QTgw.cau-4B* region, six high-confident genes were annotated according to the Chinese Spring reference genome (IWGSC, RefSeq v1.1). **f**, Comparison of the genomic sequences between Shi4185 and Heng597 within the QTL region revealed a large fragment deletion (~500 kb) spanning three high confident genes including *ZnF-B*, *EamA-B* and *Rht-B1* in Heng597. **g**, Nucleotide diversity of the genomic region spanning *ZnF*, *EamA-B* and *Rht-B1* in wild emmer (WE, 28 accessions), domesticated tetraploid (DT, 93 accessions) and domesticated hexaploid wheat materials (HEX, 289 accessions). **h**, Microcollinearity analysis of the orthologs of *ZnF*, *EamA* and *Rht-1* (or their orthologs) among different plant species. Source data

**Extended Data Fig. 2:**
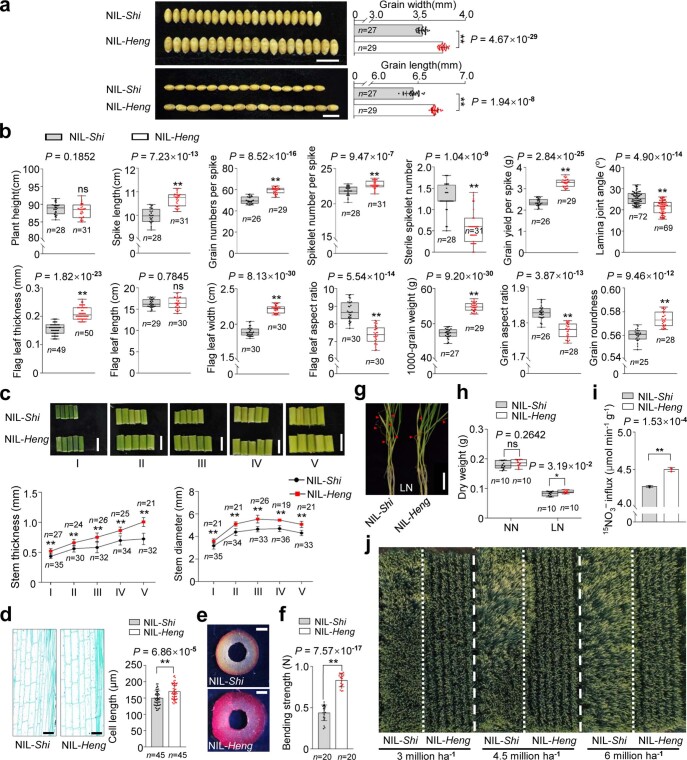
NIL-*Heng* with deleted *r-e-z* haploblock carries many favorable agronomic traits. **a**, Comparison of the grain sizes (length and width) between the near isogenic lines NIL-*Shi* and NIL-*Heng*. Scale bars = 1 cm. **b**, Comparison of spikelet number per spike, sterile spikelet number, grain yield per spike, grain roundness, flag leaf thickness, flag leaf length, flag leaf width and flag leaf aspect ratio between the NILs. **c**, Stem sections from different internodes of the two NILs. I, II, III, IV and V represent the 1^st^ to 5^th^ internodes from top to bottom, respectively. Quantitative analyses of stem thickness and stem diameter were separately performed using different internodes collected from independent wheat plants (For *P* values, see Source Data). Scale bars = 1 cm. **d**, Comparison of the length of parenchymatic cells from longitudinal sections of the fully elongated uppermost internodes between NIL-*Shi* and NIL-*Heng* at the anthesis stage (*n* = numbers of parenchymatic cells). Scale bars, 100 μm. **e**, Phloroglucinol staining of the culms from the 1^st^ internodes of NIL-*Shi* and NIL-*Heng* plants at heading stage showing the culm thickness. The experiment was repeated independently three times with similar results. Scale bars, 100 μm. **f**, Comparison of the bending strength of the 4^th^ internodes at heading stage between NIL-*Shi* and NIL-*Heng* plants. **g**, Phenotypes of NIL-*Shi* and NIL-*Heng* seedlings grown under the low nitrogen (LN, 0.5 mM KNO_3_) condition. Scale bar, 10 cm. Red triangles point to yellow leaves. **h**, Comparison of dry weight between NIL-*Shi* and NIL-*Heng* seedlings grown under low nitrogen (LN) and normal nitrogen (NN) conditions, respectively. **i**, ^15^N uptake analysis of NIL-*Shi* and NIL-*Heng* seedlings (*n* = 3 biologically independent samples). **j**, Improved lodging resistance of NIL-*Heng* compared to that of NIL-*Shi* planted in standard field plots. In **a**,**b**,**c**,**f**,**h**,*n* = numbers of biologically independent samples. In **b**,**h**, the horizontal bars of boxes represent minima, 25th percentiles, medians, 75th percentiles and maxima. Data in **a**,**c**,**f**,**d** are mean ± s.d. *P* values were calculated by a two-tailed Student’s *t*-test (** *P* < 0.01; * *P* < 0.05; ns, no significant difference). Source data

Analysis of quantitative trait loci in a segregating wheat population of Heng597 (Heng) × Shi4185 (Shi) identified a quantitative trait locus, *QTgw.cau-4B*, for higher thousand-grain weight (TGW) from Heng (Fig. 1a, Extended Data Fig. 1a–e and Supplementary Tables 1 and 2). Further gene mapping revealed that *QTgw.cau-4B* was associated with deletion of a fragment of about 500 kilobases, designated as *r-e-z*, in the Heng genome (Fig. 1a), as observed in a previous study^16^. The *r-e-z* fragment deletion resulted in the loss of three high-confidence genes, *Rht-B1, EamA-B* and *ZnF-B* (Extended Data Fig. 1f). Further genetic analysis confirmed that the genotypes with the *r-e-z* deletion showed a similar effect in shaping semi-dwarfism as the genotypes carrying the *Rht-B1b, EamA-B* and *ZnF-B* alleles (Fig. 1b and Extended Data Fig. 2b). However, the deletion of the *r-e-z* haploblock was strongly associated with higher grain weight when compared to that of the genotypes carrying the *Rht-B1b, EamA-B* and *ZnF-B* haploblock, indicating a potential application of *r-e-z* haploblock deletion in enhancing the grain yield of semi-dwarfing varieties (Fig. 1b and Extended Data Fig. 2a,b). The highly conservative genomic sequence of the *r-e-z* haploblock among wheat accessions and in other plant species (Extended Data Fig. 1g,h) indicates potentially broad applications of *r-e-z* block deletion in designing new semi-dwarf varieties of wheat and other crops.

## *r-e-z* confers desirable semi-dwarf trait

To assess the phenotypic effects of the *r-e-z* haploblock deletion, we generated a pair of near-isogenic lines (NILs) with NIL-*Heng* harbouring the *r-e-z* deletion and NIL-*Shi* carrying *Rht-B1b, EamA-B* and *ZnF-B* in chromosome 4B. Both NILs carry *Rht-D1a* in 4D and showed similar plant height, but NIL-*Heng* showed more favourable agronomic traits, including more compact plant architecture, thicker and sturdier culms, larger flag leaves and spikes, and higher grain weight than NIL-*Shi* (Fig. 1b and Extended Data Fig. 2a–f). NIL-*Heng* also showed significantly improved NUE as evidenced by its higher biomass under the low-nitrogen condition and higher NO_3_^–^ uptake rate than those of NIL-*Shi* (Fig. 1c,d and Extended Data Fig. 2g–i). The degree of the NUE improvement was positively correlated with GRF4 protein levels in NIL-*Heng* (Fig. 1e), most likely owing to reduced DELLA protein levels^2^. Overall, these improved traits conferred by the *r-e-z* deletion resemble an ideal plant architecture towards sustainable wheat production by shaping wheat plants with reduced tiller numbers, large spikes, thick and sturdy stems, and improved NUE as described in rice^3,17^.

## *r-e-z* enhances grain yield in semi-dwarf wheat

Field tests of the two NILs in preliminary field trials planted at low and high densities (1.5-m-long rows) revealed that NIL-*Heng* produced higher harvest index, grain weight and grain yield, longer spikes and better culm quality than NIL-*Shi* at both low and high planting densities (Fig. 1f,g and Extended Data Fig. 2c–f). Notably, NIL-*Heng* exhibited a higher rate of increase in grain yield per unit than NIL-*Shi* as planting density increased (about 8.4% in low density, and about 11.9% in high density; Fig. 1g), suggesting superior adaptation of NIL-*Heng* to dense planting. In standard wheat field trials, the yield of NIL-*Heng* increased 12.1%, ranging from 10.6% to 13.8% at different planting densities, compared with those of NIL-*Shi* (Fig. 1h), which illustrates great potential of using *r-e-z* deletion to enhance grain yield of semi-dwarfing varieties. Notably, severe plant lodging was found in the NIL-*Shi* plots, but not observed in the NIL-*Heng* even for the plots with high planting densities (Fig. 1h and Extended Data Fig. 2j), suggesting that use of *r-e-z* deletion may also enhance yield stability.

**Extended Data Fig. 3:**
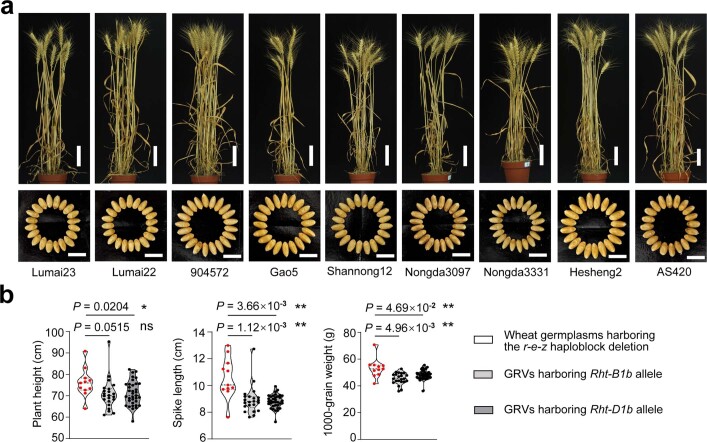
Wheat accessions harboring the *r-e-z* deletion show longer spikes and higher thousand grain weight than the ‘Green Revolution’ wheat varieties carrying *Rht-B1b* or *Rht-D1b*. **a**, The whole plants and grains of selected wheat germplasm lines harboring the *r-e-z* haploblock deletion. Scale bars are 10 cm for whole plants and 1 cm for grains. **b**, Comparison of plant height, spike length and TGW between wheat germplasms harboring the *r-e-z* haploblock deletion (n = 11 accessions) and randomly selected semidwarf varieties harboring either *Rht-B1b* (n = 22 accessions) or *Rht-D1b* (n = 35 accessions) semidominant alleles. For list of accessions, see Source Data. The bars in the violin plots represent 25th percentiles, medians and 75th percentiles. *P-*values were calculated by a two-tailed Student’s *t*-test (** *P* < 0.01; * *P* < 0.05; ns, no significant difference). Source data

Genotyping of a global collection of 556 wheat accessions identified the *r-e-z* deletion haploblock in only 12 Chinese wheat accessions (Supplementary Table 3), indicating scarcity of the *r-e-z* deletion in modern wheat. Moreover, most of these *r-e-z*-deleted wheat accessions showed significantly higher TGW and larger spikes, but similar plant height, compared to those genotypes carrying *Rht-B1b* or *Rht-D1b* alleles (Extended Data Fig. 3).

## Antagonistic effects between *ZnF-B* and *Rht-B1b*

**Fig. 2:**
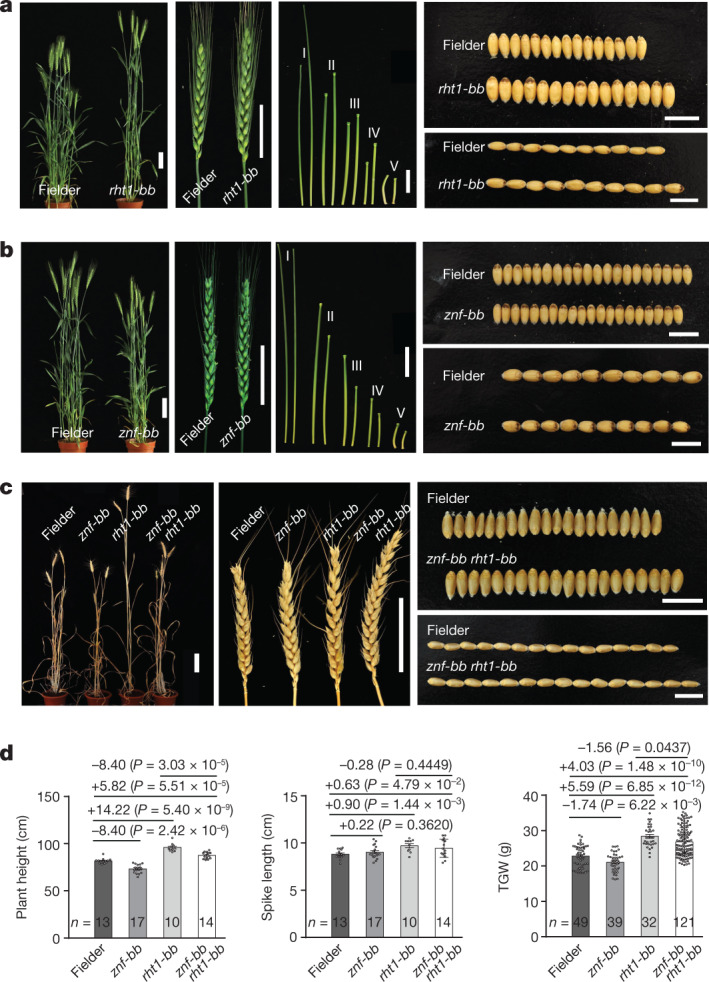
Comparison of plant height, spike length and grain yield between edited mutants and Fielder control showing the opposite effects of *Rht-B1b* and *ZnF-B* genes. **a**, The *rht1-bb* mutant had taller plants and internodes, and considerably larger spikes and grain sizes than those of Fielder. **b**, The *znf-bb* mutant had shorter plants and internodes, similar spikes and smaller grain sizes compared to those of Fielder. **c**, Comparison of plant height, spikes and grain sizes among *rht1-bb* and *znf-bb* single mutants, the *znf-bb* *rht1-bb* double mutant and the Fielder control (*n* represents the numbers of biologically independent samples). **d**, Quantification of plant height, spike length and TGW. Data are mean ± s.e.m. *P* -values were calculated by two-tailed Student’s *t*-test. Scale bars (**a**–**c**), 10 cm for whole plants, 5 cm for spikes and culms, and 1 cm for grains. I to V in **a**,**b** represent the pairs of the 1st to the 5th internodes from Fielder (left) and the mutants (right; *rht1*-*bb* in **a**, *znf*-*bb* in **b**) from top to bottom, respectively. Source data

**Extended Data Fig. 4:**
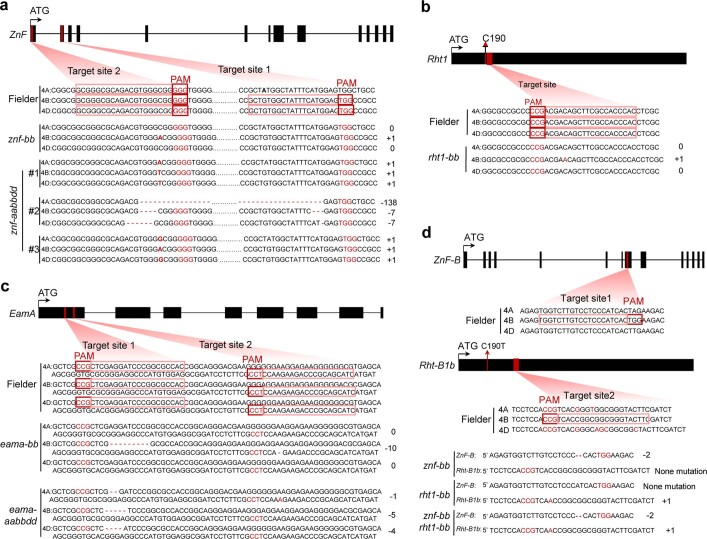
Mutations in *Rht1*, *ZnF*, and *EamA* generated by CRISPR-Cas9 gene editing. **a**–**c**, Fielder plants with mutations in *ZnF* (**a**), *Rht-B1b* (**b**), or *EamA* (**c**). **d**, Fielder plants with single or double mutations in *ZnF-B* and *Rht-B1b* generated from a single gene-editing experiment. The symbols ‘+’ and ‘−’ indicate the insertion and deletion of nucleotide, respectively, and the base numbers of insertion/deletion (bp) were shown on the right. The sgRNA target sequences were marked by pink boxes, and the PAM motifs were highlighted in red letters.

**Extended Data Fig. 5:**
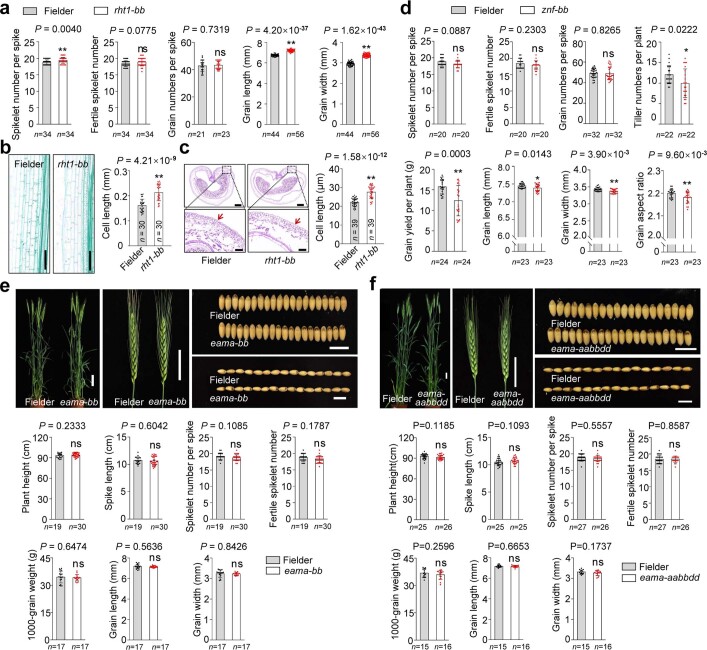
Simultaneous mutations in *ZnF-B* and *Rht-B1b* in Fielder mimic the effect of *r-e-z* deletion. **a**, Comparison of the agronomic traits including spikelet number per spike, fertile spikelet number, grain number per spike, grain length and grain width between Fielder (harboring *Rht-B1b*) and *rht1-bb* single mutant. **b**, Comparison of cell lengths of longitudinal section of the fully elongated uppermost internodes collected at anthesis stage between Fielder and the *rht1-bb* mutant (*n* = numbers of parenchymatic cells). Scale bar, 20 μm. **c**, Comparison of cell lengths of the cross sections of the developing grains collected at 10 days after pollination between Fielder and *rht1-bb* mutant (*n* = numbers of pericarp cells). Scale bars are 500 μm for the upper panels, and 100 μm for the lower panels. **d**, Comparison of the developmental and yielding traits between Fielder and *znf-bb* single mutant. **e**,**f**, Comparison of plant height, spike morphology, and grain traits between Fielder and two *Eama* mutants: *eama-bb* (**e**) and *eama-aabbdd* (**f**). In **a**,**d**,**e**,**f**, n = numbers of biologically independent samples. In **a**–**f**, data are means ± s.d.; *P* values were calculated by a two-tailed Student’s *t*-test (** *P* < 0.01; * *P* < 0.05; ns, no significant difference); Scale bars are 10 cm for whole plant, 5 cm for spike, and 1 cm for grain. Source data

To determine the gene(s) in the *r-e-z* haploblock responsible for the change in plant height and TGW, we created three independent mutants, *znf-bb*, *eama-bb* and *rht1-bb*, by gene editing of a semi-dwarf wheat variety, Fielder, to knock out *ZnF-B*, *EamA-B* and *Rht-B1b* alleles, respectively, on chromosome 4B (Extended Data Fig. 4a–c). Fielder has the same alleles at the three genes as in NIL-*Shi*, in which the *Rht-B1b* allele shows a strong suppressive effect on culm elongation and grain enlargement. The edited *rht1-bb* mutant was 14.22 cm taller and had a 5.59 g higher TGW (Fig. 2a,d and Extended Data Fig. 5a–c) whereas the z*nf-bb* mutant was 8.40 cm shorter and had a 1.74 g lower TGW than Fielder (Fig. 2b,d and Extended Data Fig. 5d). Two *EamA* mutants (*eama-bb* and *eama-aabbdd*) showed similar plant height and TGW to those of Fielder (Extended Data Fig. 5e,f). These phenotypic data strongly support that in the *r-e-z*-deleted plants, the losses of *ZnF-B* and *Rht-B1b* conferred the semi-dwarf and increased TGW, respectively. In addition, *Rht-B1b* deletion (*rht1-bb*) resulted in a marked increase in plant height, spike length and TGW, whereas *ZnF-B* deletion (*znf-bb*) led to a slight reduction in grain size and plant height with no change in spike length compared to those of Fielder (Fig. 2a,b). The *znf-bb* *rht1-bb* double mutant showed similar plant height to that of Fielder but longer spike and larger grain size than those of Fielder (Fig. 2c,d and Extended Data Fig. 4d). Therefore, the *ZnF-B* deletion confers a similar semi-dwarf trait to that of *Rht-B1b*, but less pleotropic effects on grain traits than *Rht-B1b* and has a great potential to replace the green revolution genes in semi-dwarf wheat breeding.

## ZnF is a positive regulator for BR signalling

**Fig. 3:**
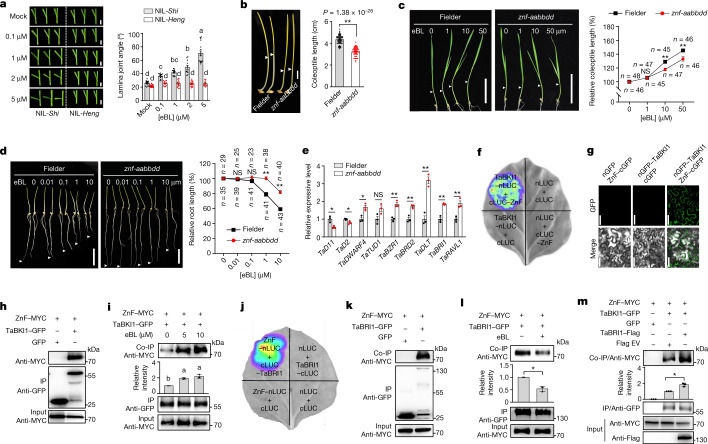
ZnF is required for BR response, and together with the TaBRI1–TaBKI1 module, gates BR signalling. **a**, BR response (to eBL) of NIL-*Shi* and NIL-*Heng* in lamina inclination assay (from 0 to 5 μM, *n* = 16, 12, 13, 11 and 17 plants for NIL-*Shi*; *n* = 19, 15, 15, 17 and 13 plants for NIL-*Heng*). **b**, Comparison of coleoptile lengths between the dark-grown *znf-aabbdd* mutant and Fielder (*n* = 54 plants). **c**,**d**, Comparison of coleoptile (**c**) and root (**d**) lengths in response to various concentrations of eBL between Fielder and *znf-aabbdd* mutant (*n* represents numbers of plants). **e**, The expression levels of BR metabolic and signalling genes in the *znf-aabbdd* mutant and Fielder measured by qRT–PCR (*n* = 3 biologically independent samples). Data in **a**–**e** are mean ± s.e.m. **f**–**h**, Interaction between ZnF and TaBKI1 confirmed by firefly luciferase (LUC) complementation imaging (**f**), bimolecular fluorescence complementation (**g**) and co-immunoprecipitation (co-IP; **h**) assays. **i**, eBL treatment enhanced ZnF–TaBKI1 interaction. **j**,**k**, Interaction between ZnF and TaBRI1 confirmed by firefly luciferase complementation imaging (**j**) and co-IP (**k**) assays. **l**, eBL treatment (5 μM) attenuated ZnF–TaBRI1 interaction. **m**, Co-IP assay confirmed that TaBRI1 enhanced the interaction between ZnF with TaBKI1. EV, empty vector. Protein levels in **i**,**l**,**m** were quantified using ImageJ software (*n* = 3 independent experiments; data are mean ± s.d.). Arrowheads in **b**–**d** indicate the tips of coleoptiles (**b**,**c**) or main roots (**d**). Different letters in **a**,**i** indicate significant differences (*P* < 0.05, one-way ANOVA, Tukey’s HSD test). In **b**–**e**,**l**,**m**, *P < 0.05; **P < 0.01; NS, not significant (two-tailed Student’s *t*-test). In **f**–**h**,**j**,**k**, all experiments were repeated independently at least twice with similar results. Scale bars, 0.5 cm (**a**), 1 cm (**b**), 5 cm (**c**,**d**) and 50 μm (**g**). Source data

**Extended Data Fig. 6:**
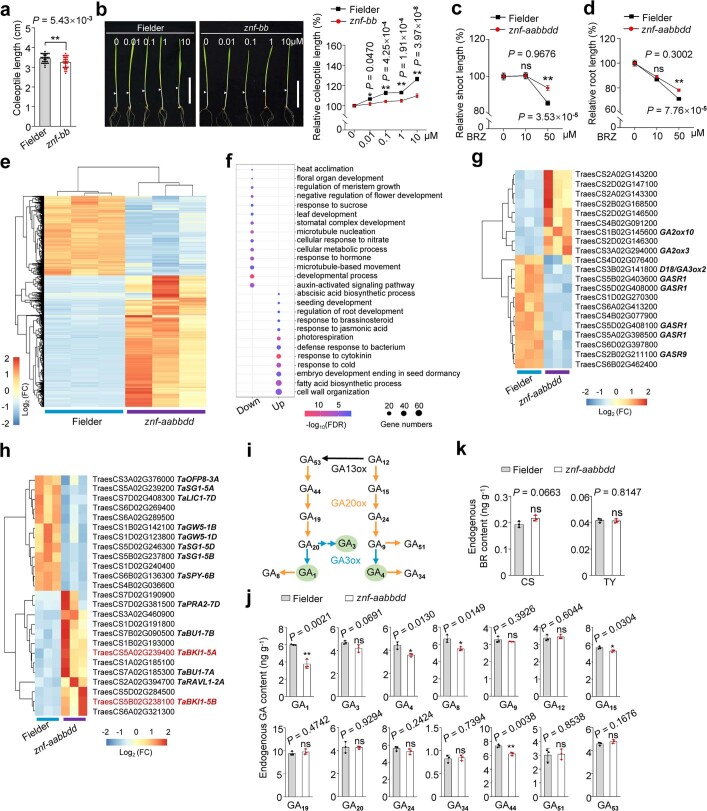
ZnF positively regulates brassinosteroid signalling. **a**, Comparison of coleoptile lengths between the *znf-bb* mutant and Fielder (*n* = 28 biologically independent samples). **b**, Comparison of relative coleoptile lengths in response to various concentrations of epi-brassinolide (eBL) treatments between Fielder and *znf-bb* mutant (From 0 to 10 μM, *n* = 45, 45, 44, 45, and 45 plants for Fielder; *n* = 48, 48, 46, 48 and 45 plants for *znf-bb* mutant). Scale bars, 5 cm. **c**,**d**, Sensitivity to brassinazole (BRZ, BR synthesis inhibitor) was reduced in the *znf-aabbdd* triple mutant compared to Fielder. The relative shoot (**c**) and root (**d**) lengths of *znf-aabbdd* mutants compared to those in Fielder treated with different concentrations of BRZ. In **c**, from 0 to 50 μM, *n* = 32, 37 and 48 plants for Fielder; *n* = 36, 35 and 55 plants for *znf-aabbdd* mutant. In **d**, from 0 to 50 μM, *n* = 28, 34 and 52 plants for Fielder; *n* = 35, 32 and 48 plants for *znf-aabbdd* mutant. Data in **a**–**d** are mean ± s.e.m. **e**, Heatmap shows the differentially expressed genes (DEGs) in a *znf-aabbdd* mutant (line 2) relative to those in Fielder. **f**, Gene Ontology (GO) analysis of the DEGs in **e**. Up stands for upregulated DEGs in the *znf-aabbdd* mutant; down stands for downregulated DEGs in *znf-aabbdd*. **g**, Heatmap shows the DEGs involving in gibberellic acid (GA) metabolism and signaling. **h**, Heatmap shows the DEGs related to BR signaling. DEGs in **e**,**g**,**h** are identified using *P* value < 0.05 and absolute Log_2_(Fold change, FC) > 1 as criteria; Colors represent log2-fold change comparing relative expression. **i**, Schematic representation of the GA metabolic pathway in higher plants. Bioactive GAs (GA1, GA3, GA4) are marked with green oval background. **j**, Comparison of the levels of two GA isoforms between Fielder and *znf-aabbdd* (#2). **k**, Comparison of the levels of castasterone (CS) and typhasterol (TY) between Fielder and *znf-aabbdd* (#2). In **j**,**k**, Data are means ± s.d. (*n* = 3 biologically independent samples); * *P* < 0.05; ** *P* < 0.01; ns, no significant difference (two-tailed Student’s *t*-test). Source data

**Extended Data Fig. 7:**
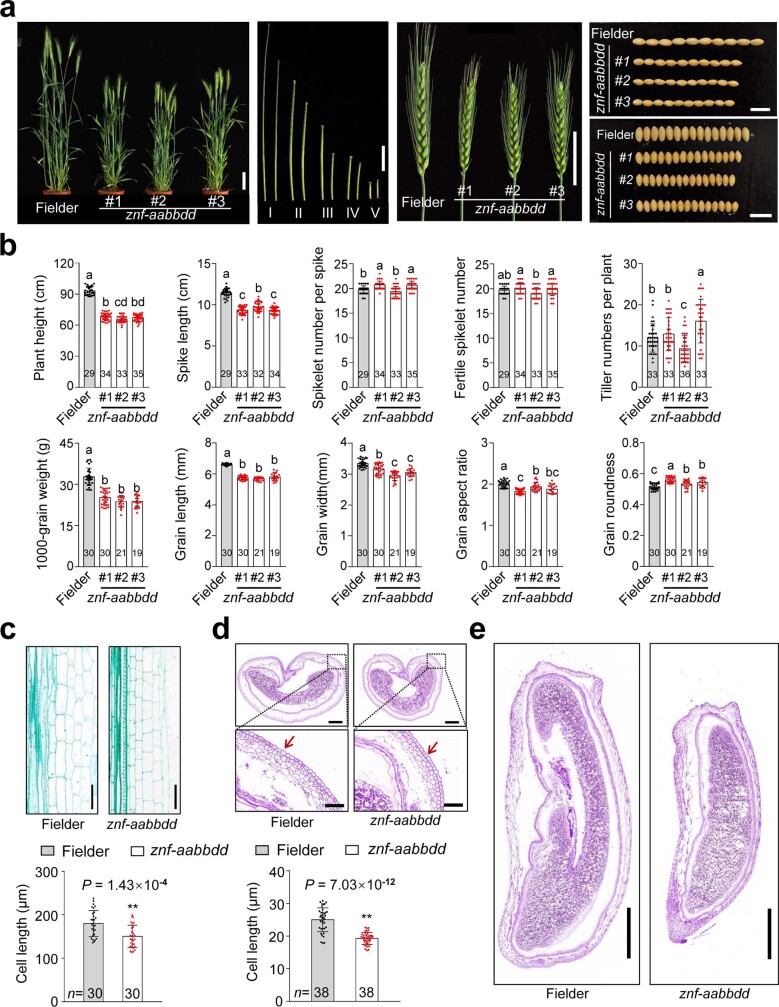
Comparison of phenotypic traits between *znf-aabbdd* triple mutants and Fielder. **a**, Pictures of whole plants, internodes, spikes and grain traits of Fielder and three independent *znf-aabbdd* mutant lines (#1, #2, and #3). Scale bars are 10 cm for whole plant, 5 cm for stem and spike, and 1 cm for grain. **b**, Statistical comparison of the phenotypic traits between Fielder and *znf-aabbdd* mutants. Different letters indicate significant differences (*P* < 0.05, one-way ANOVA, Tukey’s HSD test; for *P* values, see Source Data). **c**, Scanning micrographs of the longitudinal section of fully elongated uppermost internodes of Fielder and a *znf-aabbdd* mutant at anthesis stage. Scale bars are 200 μm. *n* = numbers of parenchymatic cells. **d**,**e**, Pericarp cell lengths from scanning micrographs of the cross section (**d**) and the longitudinal section (**e**) of the developing grains of Fielder and a *znf-aabbdd* mutant collected at 10 days after pollination (*n* = numbers of pericarp cells). *P* values in **c**,**d** were calculated by a two-tailed Student’s *t*-test (** *P* < 0.01). Scale bars in **d** are 500 μm for the upper panels, 100 μm for the lower panels; scale bars in **e** are 1 mm. Data in **b**,**c**,**d** are mean ± s.d. Source data

As *ZnF* regulates plant height, we further explored its biological functions by evaluating the phenotypic changes of the edited *ZnF* mutants. The *znf-bb* mutant produced shorter coleoptiles in the dark (Extended Data Fig. 6a), and showed much lower sensitivity to the application of epi-brassinolide (eBL, the active BR) than Fielder (Extended Data Fig. 6b), which is consistent with the observation of the BR-insensitive phenotype in NIL-*Heng* (Fig. 3a). To rule out functional redundancy from *ZnF* homoeologues, we generated *znf-aabbdd* triple mutants by knocking out all three *ZnF* homoeologues from the A, B and D subgenomes (Extended Data Fig. 4a). As expected, all of the *znf-aabbdd* mutants had significantly shorter coleoptiles in the dark and plant height, and were insensitive to eBL and brassinazole, a BR biosynthetic inhibitor (Fig. 3b–d and Extended Data Figs. 6c,d and 7). Transcriptomic profiling and quantitative PCR with reverse transcription (qRT–PCR) assays revealed significant changes in the transcripts of genes related to BR biosynthesis and signalling in *znf-aabbdd* compared with those in Fielder. These BR-related genes include *TaD11*, *TaD2* and *TaDWARF4* for cytochrome P450 enzymes, *TaBRD2* for an oxidoreductase, *TaBRI1* for BR signalling receptor, *TaTUD1* for an E3 ligase, and *TaRAVL1*, *TaBZR1* and *TaDLT* for transcription factors (Fig. 3e, Extended Data Fig. 6e,f,h and Supplementary Table 4). These results indicated that *ZnF* may act as a positive regulator for BR signalling. The epistatic interaction of BR signalling with GA biosynthesis as previously reported^11,18,19^ was also observed in the *znf-aabbdd* mutants. The bioactive GA biosynthetic gene DWARF18(D18) encoding GA3-oxidase-2 was significantly downregulated, whereas the bioactive GA deactivation genes, *GA2ox10* and *GA2ox3*, were upregulated (Extended Data Fig. 6g), resulting in a reduction in endogenous bioactive GA levels in the mutants (Extended Data Fig. 6i,j). Meanwhile, the levels of endogenous BR, including castasterone and typhasterol, were not significantly different between the *znf-aabbdd* mutants and Fielder (Extended Data Fig. 6k).

## ZnF–TaBRI1–TaBKI1 module gates BR signalling

**Extended Data Fig. 8:**
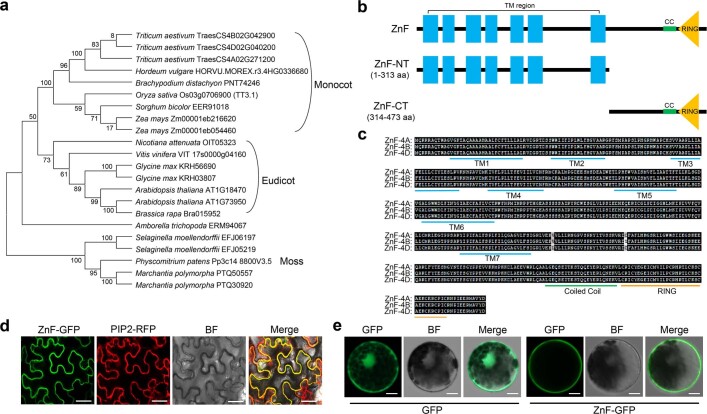
ZnF is an evolutionally conserved plasma membrane (PM)-localized protein across plant species. **a**, Phylogenetic analysis of ZnF and its orthologs in different plant species. **b**, Schematic representation of the protein structure of ZnF. Blue boxes represent the putative transmembrane (TM) domains; green boxes are coiled coil (CC) domains; yellow triangles are the RING domains in ZnF. **c**, Protein sequence alignment of the three homoeologs of ZnF proteins deduced from AA, BB, and DD subgenomes of wheat. **d**, Transient expression of ZnF-GFP fusion proteins in *N. benthamiana* leaf epidermal cells for subcellular localization of ZnF. The red fluorescent protein (RFP)-tagged PIP2 was used as the plasma membrane marker. Scale bars, 20 μm. **e**, Transient expression of ZnF-GFP fusion proteins in wheat protoplasts. Scale bars, 10 μm. In **d**,**e**, all experiment was repeated independently three times with similar results.

**Extended Data Fig. 9:**
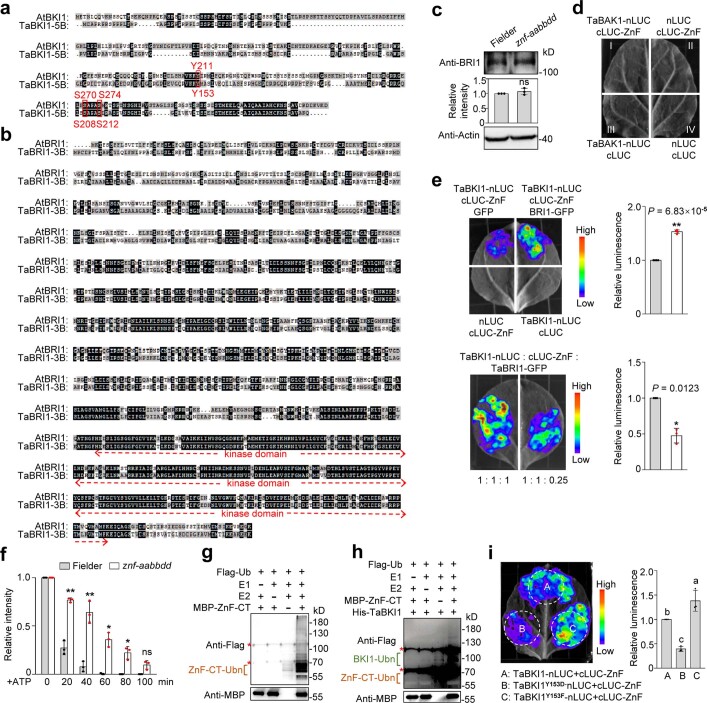
ZnF interacts with TaBRI1 and TaBKI1, and ZnF mediates TaBKI1 ubiquitination and degradation. **a**,**b**, BKI1 (**a**) and BRI1 (**b**) protein sequence alignment between *Arabidopsis thaliana* and Triticum aestivum. Point mutations in putative phosphorylation sites of BKI1 in *Arabidopsis* or wheat is marked with red boxes. The sequence of kinase domain is underlined by the red dashed-arrows. **c**, The protein levels of TaBRI1 in Fielder and a *znf-aabbdd* mutant (#2) were quantified by ImageJ software. Actin served as a loading control (*n* = 3 independent experiments). **d**, The firefly luciferase (LUC) complementation imaging (LCI) assay shows no interaction between ZnF and TaBAK1. **e**, LCI assays confirmed that the co-expression of TaBRI1 with TaBKI1 and ZnF enhanced the interaction between TaBKI1 and ZnF. The combination ratios of *A. tumefaciens* strains harboring different expression vectors were indicated (*n* = 4 independent experiments in upper panels, *n* = 3 independent experiments in lower panels). **f**, Comparison of the His-TaBKI1 degradation rates in the cell extracts between Fielder and *znf-aabbdd* (#2) in a cell-free degradation assay (*n* = 3 independent experiments). **g**, In vitro self-ubiquitination activity of the MBP-tagged ZnF-CT_314–473aa_ containing the intact RING domain. Ubn stands for ubiquitin conjugates. **h**, In vitro ubiquitination of His-TaBKI1 by ZnF-CT. Asterisk indicates the nonspecific bands. **i**, Differential interaction intensities of ZnF with TaBKI1 and its mutant forms. The wild type TaBKI1-nLUC, the mutant TaBKI1^Y153D^-nLUC (Tyr-to-Asp mutation at the 153 residue), and TaBKI1^Y153F^-nLUC (Tyr-to-Phe mutation at the 153 residue) were separately co-expressed with cLUC-ZnF, and the interaction intensities represented by LUC signals were analyzed with IndiGo software (*n* = 3 independent experiments). In **c**,**e**,**f**, asterisks indicate the significant differences (* *P* < 0.05; ** *P* < 0.01; ns, no significant difference) from two-tailed Student’s *t*-tests. Different letters in **i** indicate significant differences (*P* < 0.05, one-way ANOVA, Tukey’s HSD test). In **d**,**g**,**h**, all experiment was repeated independently at least twice with similar results. Data are mean ± s.d. (for *P* values, see Source Data). Source data

*ZnF* is an evolutionarily conserved gene across plant species and is orthologous to *Thermo-tolerance 3.1* (*TT3.1*) in rice^20^ (Extended Data Fig. 8a). ZnF harbours a coiled coil domain and a RING-finger domain in its carboxy terminus (CT) and seven transmembrane domains in its amino terminus, suggesting that ZnF is a plasma membrane (PM)-localized protein (Extended Data Fig. 8b–e). BR signalling is initially perceived by a BR receptor, BR INSENSITIVE 1 (BRI1), and a co-receptor, BRI1-ASSOCIATED RECEPTOR KINASE 1 (BAK1), on the PM, and the PM-associated protein BRI1 KINASE INHIBITOR 1 (BKI1) suppresses this perception^12,21^. To determine whether ZnF is functionally related to these PM-localized BR signalling regulators, we isolated wheat orthologues of BRI1, BAK1 and BKI1 (Extended Data Fig. 9a,b) and investigated their physical interactions with ZnF. The results confirmed that ZnF specifically interacted with TaBKI1 (Fig. 3f–h) and TaBRI1 (Fig. 3j,k), but not with TaBAK1 (Extended Data Fig. 9d). Moreover, eBL enhanced ZnF–TaBKI1 interaction (Fig. 3i), but reduced ZnF–TaBRI1 conjugation (Fig. 3l). The addition of TaBRI1 intensified ZnF–TaBKI1 interaction (Fig. 3m and Extended Data Fig. 9e). These results confirm that TaBRI1, TaBKI1 and ZnF together form a dynamic BR-responsive protein complex in which TaBRI1 facilitates the ZnF–TaBKI1 conjugation in response to BR signalling.

## ZnF degrades TaBKI1 on the PM

**Fig. 4:**
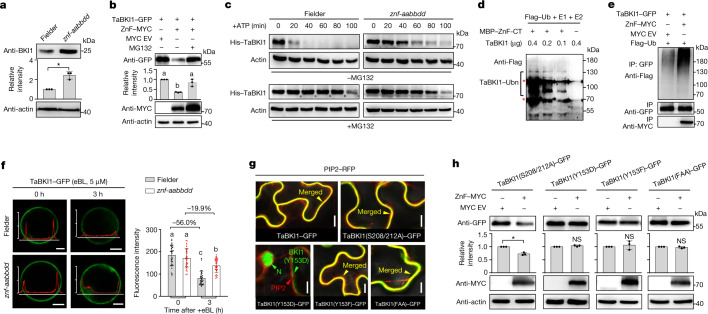
ZnF mediates TaBKI1 ubiquitylation and degradation on the PM. **a**, More TaBKI1 accumulated in the *znf-aabbdd* mutant than in Fielder. **b**, ZnF facilitates TaBKI1 degradation in *N. benthamiana*. **c**, His–TaBKI1 was degraded faster in Fielder than in *znf-aabbdd* cell extracts in a cell-free degradation assay. The results are representative of three independent experiments (Extended Data Fig. 9f). **d**,**e**, ZnF and ZnF-CT (N terminus containing an intact RING domain) both mediated ubiquitylation of TaBKI1 in vitro and in vivo. The red asterisks represent nonspecific bands. Ub, ubiquitin; Ubn, poly-ubiquitin. **f**, PM-associated TaBKI1–GFP was quickly reduced in Fielder but not in the *znf-aabbdd* protoplast cells after 3 h of exposure to eBL (*n* = 30 protoplast cells). The *y* axes in the left panels show relative intensity of GFP signal quantified by ZEN 2.3 software. Each axis label represents relative intensity of 150. **g**, Subcellular localization of TaBKI1 and its mutant forms. **h**, ZnF-mediated degradation of TaBKI1 mutant forms. Protein levels in **a**,**b**,**h** were quantified using ImageJ software (*n* = 3 independent experiments). In **d**,**e**,**g**, all experiments were repeated independently at least twice with similar results. In **a**–**c**,**h**, actin served as a loading control. In **a**,**h**, *P < 0.05; NS, not significant (two-tailed Student’s *t*-test). Different letters in **b**,**f** indicate significant differences (*P* < 0.05, one-way ANOVA, Tukey’s HSD test). In **a**,**b**,**f**,**h**, data are mean ± s.d. Scale bars, 10 μm (**f**,**g**). Source data

Most RING proteins function as E3 ubiquitin ligases to trigger protein ubiquitylation and degradation^22^. The *znf-aabbdd* mutant expressed a higher level of TaBKI1, but the same level of TaBRI1, compared to those in Fielder (Fig. 4a and Extended Data Fig. 9c), suggesting that ZnF might selectively degrade TaBKI1 in Fielder. In *Nicotiana benthamiana* cells, ZnF strongly suppressed TaBKI1 accumulation, but this was reversed after addition of a 26S proteasome inhibitor MG132 (Fig. 4b). In a cell-free degradation assay, His–TaBKI1 was degraded faster in the protein extracts of Fielder than in the *znf-aabbdd* mutant (Fig. 4c and Extended Data Fig. 9f). ZnF also ubiquitylated TaBKI1 both in vitro and in vivo (Fig. 4d,e and Extended Data Fig. 9g,h). Taken together, these results confirm that ZnF acts as an E3 ubiquitin ligase to ubiquitylate TaBKI1 for proteasomal degradation.

Previous studies demonstrated that BR can trigger rapid dissociation of BKI1 from the PM into the cytosol, which defines a crucial mechanism underlying the fast elimination of PM-associated BKI1 to activate BRI1 (refs. ^12,21,23,24^). However, eBL quickly reduced the level of PM-associated TaBKI1–GFP fusion proteins only in Fielder protoplast cells, not in the *znf-aabbdd* mutant cells (Fig. 4f), indicating that the ZnF-mediated TaBKI1 degradation is required for the reduction of PM-associated TaBKI1 in response to the BR signal. We substituted amino acids in TaBKI1 to generate constitutively PM-associated TaBKI1(S208/212A) and TaBKI1(Y153F) and constitutively PM-disassociated TaBKI1(Y153D) protein mutants^23,24^ (Fig. 4g and Extended Data Fig. 9a), and found that ZnF selectively degraded the PM-associated TaBKI1(S208/212A), but not the PM-disassociated TaBKI1(Y153D) (Fig. 4h), indicating that the ZnF-mediated TaBKI1 degradation occurs exclusively on the PM. Unexpectedly, the PM-associated TaBKI1(Y153F) was not degraded by ZnF, although a strong TaBKI1(Y153F)–ZnF interaction was detected (Extended Data Fig. 9i). The same is true for TaBKI1(FAA) with Y153F and S208/212A substitutions (Fig. 4h). These results indicate that an intact Y153 (Y211 in *Arabidopsis* BKI1) is essential for TaBKI1 degradation.

## Application of *r-e-z* deletion in wheat breeding

**Fig. 5:**
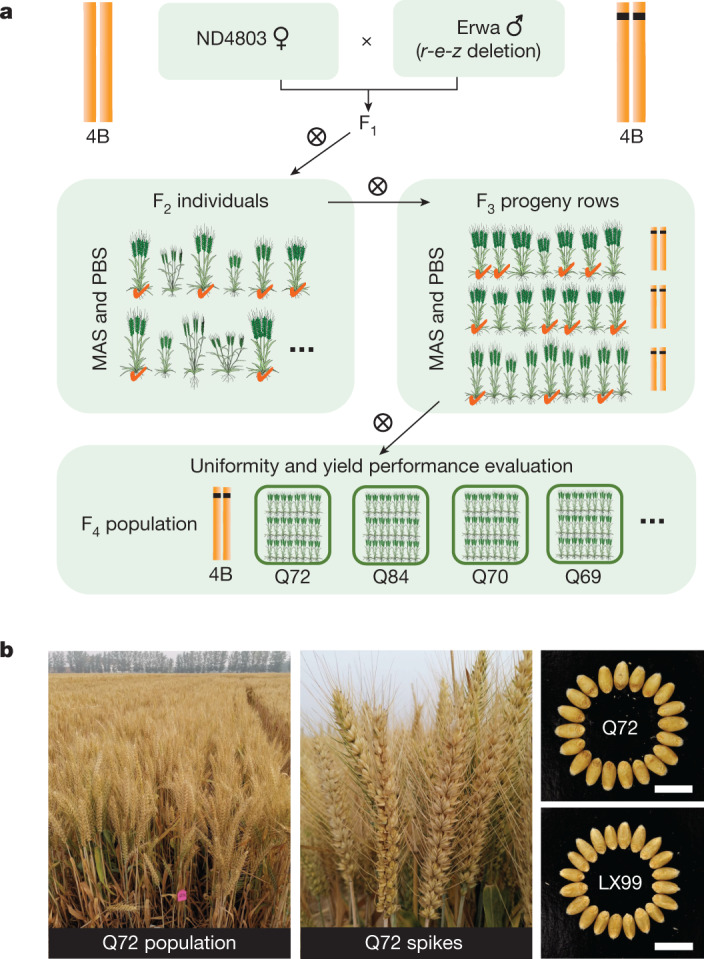
Application of *r-e-z* haploblock deletion in high-yield semi-dwarf wheat breeding. **a**, Scheme for breeding high-yield semi-dwarf wheat varieties harbouring the *r-e-z* deletion haploblock. MAS, marker-assisted selection; PBS, phenotype-based selection. **b**, Field population and spikes of a selected line, Q72, and a comparison of its grain size with that of a high-yield GRV, LX99. Scale bars, 1 cm.

To introduce the *r-e-z* haploblock deletion into the GRVs that are grown at present in commercial production to obtain new semi-dwarf wheat varieties with enhanced grain yields, we crossed Nongda4803 (ND4803) harbouring *Rht-B1b* and wild-type *Rht-D1a* and Erwa carrying the *r-e-z* deletion and wild-type *Rht-D1a* and selected the *r-e-z* deletion block using markers and other traits using conventional phenotypic selection methods (Fig. 5a) in the breeding population. Finally, we successfully selected four lines (Q69, Q70, Q72 and Q84) with desirable plant height and yield. In a field trial, these lines showed yield increases of 6.48% to 15.25% compared to the control Liangxing99 (LX99), a *Rht-D1b* high-yielding variety widely grown in China with cumulative planting area exceeding 5 million hectares (Fig. 5b and Table 1). The yield increase in these *r-e-z*-introgression lines was mainly attributed to marked increase in grain number per spike and TGW in comparison with the LX99 control, although the *r-e-z*-introgression lines had lower spike number per unit area than LX99 (Table 1), revealing different yield component profiles between the *r-e-z*-introgression lines and traditional GRVs. Taken together, these findings illustrate that our newly designed wheat breeding system that uses the *r-e-z* haploblock deletion to achieve semi-dwarfism not only effectively reduces plant height like *Rht-B1b*, but also increases yield potential and sustainability of wheat production.

**Table 1:** Field yield evaluation of the four selected lines at the population level

Line	TGW (g)	GNS	SNPA	Grain yield (kg ha^−1^)
LX99	42.8	30.8	615.0	10,331
Q72	54.5 (+11.7)	45.1 (+14.3)	445.5 (−169.5)	11,906 (+15.2%)
Q84	48.2 (+5.4)	49.0 (+18.2)	460.5 (−154.5)	11,717 (+13.4%)
Q70	48.8 (+6.0)	41.1 (+10.3)	435.0 (−180.0)	11,285 (+9.23%)
Q69	52.3 (+9.5)	42.0 (+11.2)	439.5 (−175.5)	11,000 (+6.48%)

Statistical analysis of the main yield components and final grain yields of the four selected lines with LX99 as the control in the field. GNS, grain number per spike; SNPA, spike number per unit area.

## Discussion

Since the 1960s, the NUE-repressing alleles (*Rht-B1b* and *Rht-D1b*) have been present in almost all commercially grown wheat varieties worldwide, which has created a substantial challenge to global sustainable wheat production due to increased N fertilizer input requirement^2,5,25,26^. In this study, we identified a natural *r-e-z* haploblock deletion that results in the loss of three genes, *Rht-B1*, *EamA-B* and *ZnF-B*. Compared to *Rht-B1b* lines, the lines with *r-e-z* haploblock deletion conferred the same semi-dwarf trait, but with considerably higher NUE, more compact plant architecture, larger spikes and grains, higher grain yields, and a more stable population suitable for dense planting (Fig. 1c–h and Extended Data Fig. 2). The higher accumulation of GRF4 protein and lower abundance of DELLA protein in NIL-*Heng* harbouring the *r-e-z* deletion than in NIL-*Shi* carrying the *Rht-B1b* allele (Fig. 1e) suggested an antagonistic interaction between GRF4 and DELLA^2^. Notably, this *r-e-z* haploblock deletion is very rare in modern wheat accessions, and thereby can be readily deployed into new wheat varieties to break the grain yield ceiling resulting from widespread application of the green revolution alleles, as demonstrated in this study (Fig. 5 and Table 1).

**Extended Data Fig. 10:**
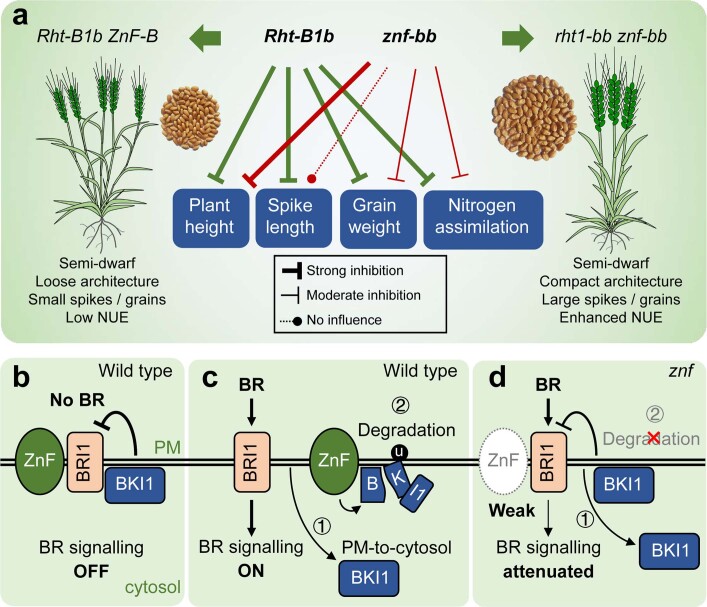
A proposed working model summarizing highly divergent effects of *Rht-B1* and *ZnF* in regulating different wheat agronomic traits, and a pivotal role of ZnF in regulating BR signalling. **a**, A schematic representation to illustrate how a combination of ‘Green Revolution’ *Rht-B1b* and *ZnF-B* regulates wheat plant architecture, nitrogen use efficiency, and grain yield traits. *Rht-B1b* allele strongly reduces plant height, spike length, grain size, and nitrogen assimilation. However, the loss of *ZnF-B* also reduces plant height as the ‘Green Revolution’ genes, but shows only marginal or undetectable reduction in spike length and grain size. **b**–**d**, A proposed model to illustrate what different *ZnF* alleles do to regulate BR signalling. **b**, The plasma membrane (PM) associated BKI1 interacts with the BR receptor BRI1 to repress its activity in the absence of BR; **c**, The PM-associated BKI1 proteins are disassociated from the PM to cytosol, or degraded by ZnF directly on the PM upon the perception of BR signal to ensure the activation of BRI1 and BR signalling; **d**, In a *znf* mutant, the PM-associated BKI1 is partially eliminated, leading to partial attenuation of BR signal that retarded plant growth. Red ‘ × ’ refers to the blockage of BKI1 degradation. In summary, we discovered that a natural *r-e-z* haploblock deletion that caused the loss of *Rht-B1*, *EamA-B*, and *ZnF-B* confers reduced plant height, and increased grain weight and yield. Genetic analysis shows that the deletion of both *Rht-B1* and *ZnF-B* is essential for the improvement of these traits in wheat. ZnF acts as an activator of BR signal, a different mechanism from the DELLA proteins encoded by well-known *Rht-B1* that act as GA signalling repressors. The *ZnF-B*–*Rht-B1* combination is functionally independent but genetically linked genetic unit to balance BR–GA crosstalk in wheat. Based on our findings, we propose a new semidwarf breeding strategy to use the deletion of both *Rht-B1* and *ZnF-B* to design new semidwarf wheat varieties with more compact wheat plant architecture, largely improved culm quality, enhanced NUE, and increased grain yields than the traditional green revolution wheat varieties.

The data from gene editing of Fielder demonstrated that the divergent roles of the *r-e-z* deletion in reducing plant height and increasing grain weight and NUE are attributed to the combined effects of deletion of both *Rht-B1* and *ZnF-B* (Fig. 2c), thus defining the two neighbouring genes as an integral genetic unit for fine-tuning multiple agronomic and yield traits in wheat (Extended Data Fig. 10). Unlike the gain-of-function *Rht-B1b* (or *Rht-D1b*) allele that strongly represses not only culm elongation but also spike and grain development and NUE^2,5,25^ (Fig. 2a,c,d), *ZnF* is probably a plant height-regulating gene whose null allele (illustrated by *znf-bb*) confers a semi-dwarfing effect with no or little undesired pleotropic effects on other agronomic traits (Fig. 2b–d and Extended Data Fig. 10a). Thus, we propose a new strategy to redesign semi-dwarf varieties by deleting the widely used *Rht-B1b* to overcome the growth defect and yield penalties caused by this green revolution allele and *ZnF-B* to retain the semi-dwarf statures. This can be achieved through genetic engineering such as the genotype-independent CRISPR–Cas9-based multigene-editing strategy in wheat^27,28^.

Mechanistically, the semi-dwarf trait conferred by *ZnF* deletion is due to BR signalling deficiency, which is largely different from the traditional GA-insensitive semi-dwarfism induced by *Rht-B1b* or *Rht-D1b*. At a molecular level, ZnF acts as an E3 ligase to specifically target TaBKI1 for proteasomal degradation, and thus facilitates BR perception (Extended Data Fig. 10b,c); loss of *ZnF* dampens the BR-triggered TaBKI1 elimination from the PM, leading to a BR-deficient semi-dwarfism (Extended Data Fig. 10d). This *ZnF*-mediated regulation of BR signalling should be highly conserved across monocots and dicots, and further work will elucidate *ZnF* gene functions in other crops such as rice and maize. Thus, we may expand the application of *ZnF* as a new source of semi-dwarfing genes to breed new high-yielding varieties with desired plant height by reducing BR signalling in different crops. In summary, our study not only provides a new strategy to improve GRVs by engineering a functionally independent but genetically linked ZnF–DELLA genetic factor for sustainable agriculture, but also reveals a vital molecular mechanism of full degradation of the PM-localized BR receptor for effective activation of BR signalling.

## Methods

### Plant materials and growth conditions

An F_2_ population of 286 plants was initially generated from a cross between a low-TGW parent, Shi4185 (Shi), and a high-TGW parent, Heng597 (Heng), and used to identify *QTgw.cau-4B* for TGW. To finely map *QTgw.cau-4B*, phenotypic and marker screening of the recombinants from F_3_ to F_7_ generations, coupled with phenotypic evaluation of the progenies, identified a key residual heterozygous line, R4, that showed heterozygosity within the interval of *QTgw.cau-4B*. NILs contrasting at the quantitative trait locus (QTL) region were isolated by selfing of R4. NIL-*Shi* contains the wild-type *ZnF-B* and *EamA-B* as well as the dominant *Rht-B1b* in its B genome, whereas NIL-*Heng* lacks *Rht-B1*, *EamA-B* and *ZnF-B* genes owing to a natural deletion of a *r-e-z* haploblock of about 500 kilobases. Both NILs contain wild-type *Rht-D1a* in their D genome. A worldwide collection of 556 wheat accessions was screened for the presence of the *r-e-z* haploblock. A wheat variety, Fielder, carrying the *Rht-B1b*, *EamA-B* and *ZnF-B* alleles in its B genome and *Rht-D1a* in its D genome as in NIL-*Shi* was used for gene editing.

*N. benthamiana* plants were grown under a 16 h of light and 8 h of dark photoperiod at 23 °C, and the T_0_ and T_1_ transgenic wheat plants were grown under 16 h of light at 24 °C and 8 h of dark at 16 °C both in a greenhouse at China Agricultural University. The F_2_ segregating population was space planted in a field at the China Agriculture University Experimental Station (Beijing, People’s Republic of China) in the 2014–2015 growing season. The T_2_ and T_3_ transgenic plants were planted in a 1.5-m single-row plot spaced 0.3 m apart with 25 seeds per row in the same location in 2019 and 2020, respectively. The NILs and selected segregating families from F_3_ to F_7_ generations were planted at the China Agriculture University-Jize Experimental Station (Jize county, Handan city, Hebei province).

NIL-*Shi* and NIL-*Heng* were evaluated for agronomic traits in the field at the China Agriculture University-Jize Experimental Station. The preliminary yield trial was conducted in the 2021–2022 growing season. Two NILs were hand planted in 30-row plots of 1.5 m in length with 90 seeds per row. The space between each row was 0.3 m for low-density and 0.15 m for high-density planting, with nine replicates. The two NILs were also planted in standard yield trials in 1.2 × 7 m plots using a planter in 2022. The experiment used a paired-plot design with three planting densities and ten replicates.

Breeding lines Q69, Q70, Q72 and Q84 containing the *r-e-z* haploblock deletion and *Rht-D1a* allele were selected in a field experiment at the National Observation and Research Station of Agriculture Green Development (Quzhou county, Handan city, Hebei province, People’s Republic of China). Two elite breeding lines, Nongda4803 (ND4803, harbouring *Rht-B1b* and wild-type *Rht-D1a* as the female parent) and Erwa (with *r-e-z* deletion and wild-type *Rht-D1a* as the male parent) were used to develop the breeding population. During the 2018–2019 growing season, we phenotyped and genotyped more than 2,000 F_2_ plants and obtained 91 outstanding plants carrying the combination of *r-e-z* block deletion and desirable agronomic traits. The selected individuals were further selfed to generate independent F_3_ progeny, and phenotyping and genotyping of the F_3_ lines identified five lines with the *r-e-z* block deletion and uniform appearance in the 2019–2020 season. The selected F_3_ rows were bulk harvested to form F_4_ for uniformity and yield performance evaluation in 2020–2021 field plots by planting them in a standard field trial with 1.2 × 7 m plots at a planting density of 3.3 million seedlings per hectare. A high-yielding GRV, LX99, was used as the yield control.

### Field trait evaluation

Wheat seeds were randomly sampled from preliminary yield trials and standard field trials to measure TGW, grain length, grain width, grain aspect ratio and grain roundness using a Wanshen SC-G seed detector (Hangzhou Wanshen Detection Technology). The other agronomic traits including spike length, grain numbers per spike and flag leaf morphology were measured manually before harvest in the field. A digital dynamometer (YLK-500, ELECALL) was used to measure the bending strength of the fourth internode (from top to bottom). To assess the final yields of the NILs in either preliminary yield trials or standard field trials under different planting density, one 1-m^2^ area (1 × 1 m) was randomly selected in each plot, and all wheat plants within the selected area in the plot were harvested. Before harvesting, the plants outside the selected 1-m^2^ area were removed to avoid margin effects.

For field trait evaluation of *r-e-z*-carrying breeding lines, the plants within the standard field plots were all harvested for final yield evaluation. TGW was calculated from 3 randomly selected samples per plot with 500 grains in each sample. Grain number per spike was counted manually from 3 randomly selected replicates of 20 main spikes in each plot. Spike number per unit area was assessed by counting all of the spikes within a randomly selected 1-m-long row, and 3 replicates in each plot were carried out.

### QTL mapping and gene cloning

Single sequence repeat markers were screened in the F_2_ population of Heng × Shi to map the QTLs for grain traits. One major QTL (*QTgw.cau-4B*) for TGW, grain length and grain width was located on the short arm of chromosome 4B and three single sequence repeat markers were mapped within the interval of *QTgw.cau-4B*. Further genotypic and phenotypic analyses of the F_3_-derived residual heterozygous line mapped *QTgw.cau-4B* to the interval between the markers SNP-5 and SNP-7. The QTL explained 74.65% of the phenotypic variance. Recombinants between the flanking markers of *QTgw.cau-4B* were continuously screened from F_4_ to F_7_ generations for fine mapping, and phenotypic and genotypic data from the recombinants narrowed the QTL interval to the region between the markers M7 and ID-51 where only six high-confidence genes were annotated on the basis of RefSeq v1.1 (2018) produced by the International Wheat Genome Sequencing Consortium. Both Shi and Heng were resequenced, and their genomic sequences in the QTL region were compared to identify sequence polymorphisms. The primers used for map-based cloning are listed in Supplementary Table 5.

### Plasmid construction

For the firefly LUC complementation imaging (LCI) assay, the full-length coding sequences (CDSs) of the candidate genes, including *ZnF*, *TaBKI1*, *TaBRI1* and *TaBAK1*, were separately cloned into the *pCAMBIA1300-nLUC* and *pCAMBIA1300-cLUC* vectors through In-Fusion PCR cloning system (CL116, Biomed). For the bimolecular fluorescence complementation (BiFC) assay, CDSs for *TaBKI1* and *ZnF* were cloned into the *pEarleygate201* and *pEarleygate20*2 vectors using a Gateway cloning system (12535029, Invitrogen). For the co-IP assay, the *ZnF–MYC*, *BKI1–GFP*, *BRI1–GFP* and *BRI1–Flag* constructs were generated by inserting the CDSs of these genes into *pCAMBIA1300* vectors fused with different tag sequences (MYC, GFP and Flag) using an In-Fusion PCR Cloning kit (CL116, Biomed). To generate *His–BKI1* and *MBP–ZnF-CT* (314–473 amino acid) constructs, we used *pCold-TF* (fusing with His tag; Takara) and *pMAL-c2X* (fusing with maltose binding protein tagged) vectors. All of the primer sequences are listed in Supplementary Table 6.

### CRISPR–Cas9-mediated gene editing

The CRISPR–Cas9-based gene editing was used to knock out target wheat genes. The single guide RNA (sgRNA) target sequences were designed according to the exon sequences of the target genes using the online software E-CRISPR (http://www.e-crisp.org/E-CRISP/). The *MT1T2* vector was amplified using the primers containing sgRNAs and then cloned into the CRISPR–Cas9 vector pBUE411. The generated vector was further transformed into the Fielder variety following the *Agrobacterium tumefaciens* (strain EHA105) gene transformation procedure^29^. Subgenome-specific primer pairs were designed for mutation analysis and further screening of homozygous T_2_ and T_3_ mutant lines (Supplementary Table 6).

### eBL and brassinazole treatment

eBL (E1641, Sigma) and brassinazole (BRZ; B2829, TCI) were separately prepared by dissolving them in dimethylsulfoxide (DMSO). For eBL or BRZ treatment, 2-day-old wheat seedlings of Fielder and a *znf-aabbdd* mutant (line 2) were soaked in defined concentrations of eBL or BRZ water solution. The same volume of DMSO (blank solvent) was used as a mock control. The lengths of coleoptiles and roots were determined 7 days after the treatments. For the lamina joint inclination assay in response to eBL treatment, 1.5-cm-long leaf segments containing lamina joints were excised from 14-day-old seedlings of NIL-*Shi* and NIL-*Heng*, and incubated in eBL solutions at different concentrations in the dark for 2 days. The lamina joint angles were determined by ImageJ software (https://imagej.net/ij/). All experiments were repeated three times.

### Histological analysis

The middle part of the first internode (from top to bottom) at the heading stage (emergence of inflorescence completed at Zadoks stage 58) and developing grain at 10 days after pollination^30^ were collected to determine cell size. The collected samples were fixed in an FAA solution (10% (v/v) formaldehyde, 50% (v/v) alcohol, 5% (v/v) acetic acid and 35% (v/v) water) overnight at 4 °C, and then were embedded in paraffin, dehydrated and decolourized as described previously^31^. The samples were then cut into 4-µm-thick cross-sections using a Leica Ultracut rotary microtome (Leica Biosystems), and stained with periodic acid Schiff or 1% sarranine and 0.5% fast green (G1031, Servicebio). Photographs were taken with a microscope imaging system (DS-U3, Nikon) and the cell lengths were measured with CaseViewer 2.3 (3DHISTECH).

### qRT–PCR and RNA-sequencing assays

For the qRT–PCR assay, total RNA was extracted from wheat tissues using a TRIzol reagent (Thermo Fisher Scientific) following the manufacturer’s instructions. After the removal of genomic DNA, cDNAs were synthesized using a Reverse Transcription kit (R223, Vazyme). Real-time PCR was carried out using the SYBR Green PCR Master Mix (Q121, Vazyme) in a CFX96 Real-Time PCR System (Bio-Rad). *β*-*ACTIN* was used as the internal gene control. Each experiment was repeated three times. The primers used for qRT–PCR assays are listed in Supplementary Table 6.

For RNA-sequencing analysis, the stem samples were collected at the jointing stage (second node detectable at Zadoks stage 32)^30^, and total RNAs were extracted using TRIzol reagent. The cDNA libraries were constructed using Poly-A Purification TruSeq library reagents (Illumina), followed by sequencing on an Illumina 2500 platform. After cleaning up raw sequence reads, the clean reads were mapped to the wheat reference genome (International Wheat Genome Sequencing Consortium, RefSeq v1.1) using TopHat2 software^32^. The differentially expressed genes were analysed using the DESeq2 R package. Significant differentially expressed genes were determined using a standard procedure including adjusted *P* value (false discovery rate < 0.05) and fold change ratio (log_2_[FC] ≥ 1). Gene ontology enrichment was carried out using the online tool Triticeae-GeneTribe^33^.

### Immunoblotting and co-IP assays

Total proteins were extracted using a lysis buffer (50 mM Tris-HCl pH 7.5, 150 mM NaCl, 5 mM EDTA at pH 8.0, 0.1% Triton X-100, 0.2% NP-40, 0.6 mM phenylmethylsulfonyl fluoride (PMSF)) supplemented with a freshly added protease inhibitor cocktail (Roche LifeScience) and ΜG132 (10 μM). For the immunoblotting assay, the protein samples were separated on 10% SDS–PAGE and detected by antibodies including anti-GFP (1:2,000 dilution, ab32146, Abcam), anti-BRI1 (1:1,000 dilution, *Setaria italica* BRI1)^34^, anti-TaBKI1 (1:1,000 dilution, prepared in this study, ABclonal), anti-SLR1 (1:1,000 dilution, A16279, ABclonal) and anti-GRF4 (1:1,000 dilution, A20348, ABclonal). For the co-IP assay, about 20 μl anti-GFP magnetic agarose beads (gtma-20, Chromotek) was incubated with protein samples for 3 h at 4 °C. The beads were cleaned four times with a wash buffer (50 mM Tris-HCl pH 7.5, 150 mM NaCl, 5 mM EDTA pH 8.0, 0.6 mM PMSF and 1× protease inhibitor cocktail), and the immunoprecipitated proteins were separated by SDS–PAGE and detected with anti-GFP and anti-MYC (1:2,000 dilution, CB100002M, California Bioscience) antibodies.

### Antibody preparation

To create the anti-TaBKI1 antibody, a peptide fragment, N-EGRDDTAGKAEEDRK-C, corresponding to amino acids 121–135 of TaBKI1 was artificially synthesized and purified, and then conjugated with the keyhole limpet haemocyanin carrier before generation of the anti-TaBKI1 antibody in a rabbit.

### LCI and BiFC assays

The LCI and BiFC assays were carried out in *N. benthamiana* leaves. In brief, the nLUC and cLUC derivatives, or the nGFP and cGFP derivatives, were transformed into the *A. tumefaciens* strain GV3101. The obtained *Agrobacteria* cells harbouring the constructs were co-infiltrated into *N. benthamiana* leaves. The LUC activity was analysed 48 h after infiltration using Night SHADE LB985 (Berthold), and the fluorescence signal of GFP was observed 48 h after infiltration under a confocal microscope (LSM880, Zeiss).

### Protein subcellular localization

To localize ZnF proteins in a cell, the *35S::ZnF–GFP* and *35S::PIP2–RFP* expression vectors were separately transformed into the *A. tumefaciens* strain GV3101, and then were co-expressed in the leaf epidermal cells of *N. benthamiana*. The GFP fluorescence signal was detected about 48 h after infiltration by a confocal microscope (LSM880, Zeiss). For the assays using protoplast cells, wheat protoplasts were initially isolated from the first leaf of 7-day-old seedlings, and then the *35S::ZnF–GFP* or *35S::TaBKI1–GFP* expression vectors were separately transferred into protoplast cells following the protocol described previously^35^. The GFP fluorescence signal was detected 16 h after the transformation by a confocal microscope (LSM880, Zeiss).

### In vitro and in vivo ubiquitylation assay

The in vitro ubiquitylation assay was carried out as described previously^36^. In brief, the MBP–ZnF-CT and His–-BKI1 recombinant proteins were expressed and purified from *Escherichia coli* strain BL21 (DE3). MBP–ZnF-CT alone, or together with His–BKI1, was incubated with E1 (UBA1–GST, 50 ng), E2 (UBC10–GST, 50 ng) and Flag–ubiquitin (1 µg) in a reaction buffer (50 mM Tris-HCl pH 7.5, 5 mM ATP, 20 mM MgCl_2_ and 1 mM dithiothreitol) at 30 °C for 1.5 h. Similarly, for the assay of ZnF-mediated TaBKI1 ubiquitylation, TaBKI1 at different concentrations was incubated with MBP–ZnF-CT, E1 (UBA1–GST, 50 ng), E2 (UBC10–GST, 50 ng) and Flag–ubiquitin (1 µg) in reaction buffer. The reaction was stopped by adding SDS loading buffer. The obtained samples were boiled at 100 °C for 7 min, and the proteins were detected with anti-Flag (1:2,000 dilution, F1804, Sigma) antibody using SDS–PAGE. For the in vivo ubiquitylation assay, TaBKI1–GFP, ZnF–MYC and Flag–ubiquitin were co-expressed in *N. benthamiana* leaf epidermal cells. TaBKI1–GFP proteins were immunoprecipitated and purified to ubiquitin-conjugated TaBKI1–GFP through immunoblotting with anti-Flag (1:2,000 dilution, F1804, Sigma) antibody using anti-GFP magnetic agarose beads (gtma-20, Chromotek).

### Cell-free degradation assays

Total proteins were extracted from 2-week-old wheat seedlings with native buffer (50 mM Tris-MES pH 8.0, 0.5 M sucrose, 1 mM MgCl_2_, 10 mM EDTA, 5 mM dithiothreitol, 1 mM PMSF and 1× protease inhibitor cocktail)^37^. The protein extracts of Fielder or the *znf-aabbdd* mutant were mixed with purified His–BKI1 fusion protein in the presence or absence of 50 µM MG132. The samples incubated at room temperature (25 °C) were collected at designated time points, followed by the addition of 2× SDS loading buffer to stop the reaction. The proteins were detected by SDS–PAGE using anti-His (1:2,000 dilution, BE2017, EASYBIO) antibody.

### Phylogenetic, genetic diversity and micro-collinearity analyses

The protein sequences of ZnF and its orthologues from different plant species were extracted from the EnsemblPlants database (http://plants.ensembl.org/index.html). The phylogenetic tree was constructed using a maximum-likelihood method in the MEGA5.0 program with bootstrap (500 replicates) and complete deletion. Wheat accessions including 28 wild emmer accessions, 93 domesticated tetraploid wheat accessions and 289 hexaploid wheat accessions (Supplementary Table 7) were used for the nucleotide diversity analysis of the *Rht-B1*, *EamA-B*, *ZnF-B* gene cassette and its flanking regions using VCFtools (v0.1.13) with >100-kilobase sliding windows in 100-kilobase steps. The online tool Triticeae-GeneTribe was used for micro-collinearity analysis of the *Rht-B1*, *EamA-B*, *ZnF-B* gene cassette among different crop species^33^.

### Hydroponic cultivation for low-nitrogen treatment

The wheat seeds were initially germinated on wet filter papers. About 3 days later, the seedlings were transferred to hydroponic culture (2.5 mM KNO_3_, 0.3 mM NaH_2_PO_4_·2H_2_O, 1 mM CaCl_2_·2H_2_O, 1 mM MgSO_4_·7H_2_O, 0.77 µM ZnSO_4_·7H_2_O, 0.32 µM CuSO_4_·5H_2_O, 40 µM EDTA–FeNa·3H_2_O, 9 µM MnSO_4_·4H_2_O, 0.05 µM (NH_4_)_6_·Mo_7_O_24_·4H_2_O and 20 µM H_3_BO_3_, pH 5.8) for further cultivation. In the low-nitrogen treatment, 0.5 mM KNO_3_ was added into the nutrient solution, and the K concentration was adjusted by KCI to 2.5 mM. Six weeks after cultivation, wheat plants were harvested for phenotypic evaluation, including dry weight and fresh weight. All plants were grown in a phytotron under 16 h of light at 24 °C and 8 h of dark at 20 °C with about 70% relative humidity.^+^

### ^15^N uptake rate analysis

For ^15^N uptake analysis, wheat seedlings were cultured in the hydroponic culture (supplemented with 2.5 mM KNO_3_, pH 5.8) for two weeks. After N starvation by culturing the seedlings in a hydroponic solution without N for 2 days, wheat roots were treated with K^15^NO_3_ (98 atom% ^15^N; SigmaAldrich, number 335134) for 30 min. After washing with 0.1 mM CaSO_4_ solution and deionized water as described previously^38^, roots of seedlings were collected and dried at 70 °C for 3 days. After grinding the sample into powder, the ^15^N content in the root was measured using an isotope ratio mass spectrometer (Thermo Finnigan Delta Plus XP; Flash EA 1112) with three biological replicates.

### Endogenous phytohormone quantification

The stem tissues of the indicated wheat materials were collected at the early jointing stage (second node detectable at Zadoks stage 32)^30^, and were immediately frozen in liquid nitrogen. The quantification of endogenous GAs and BRs levels was carried out as reported previously^39^. In brief, 200 mg of the ground plant material powder was extracted with 90% aqueous methanol. Simultaneously, each of the D-labelled GA and BR compounds was added to the extraction solvents as internal standards for quantification. After effective pretreatment, GA and BR analysis was carried out on a quadrupole linear ion trap hybrid mass spectrometer (QTRAP 6500, AB SCIEX) equipped with an electrospray ionization source coupled with an ultrahigh-performance liquid chromatography instrument (Waters).

### Reporting summary

Further information on research design is available in the Nature Portfolio Reporting Summary linked to this article.

## Online content

Any methods, additional references, Nature Portfolio reporting summaries, source data, extended data, supplementary information, acknowledgements, peer review information; details of author contributions and competing interests; and statements of data and code availability are available at 10.1038/s41586-023-06023-6.

## Supplementary information

Supplementary Fig. 1 Uncropped gel images.

Reporting Summary

Supplementary Tables This file contains Supplementary Tables 1–7.

## Data Availability

The raw sequence data generated by this research have been deposited in the National Center for Biotechnology Information under the accession number PRJNA852953 for RNA sequencing. The raw sequence data of previously published resequenced accessions used in this study are available in the Sequence Read Archive under the accession codes PRJNA597250, PRJNA439156, PRJNA663409, PRJNA596843 and PRJNA544491. All other data are available in the main text or the Supplementary Information. Source data are provided with this paper.

## Source data

Source Data Fig. 1
Source Data Fig. 2
Source Data Fig. 3
Source Data Fig. 4
Source Data Extended Data Fig. 1
Source Data Extended Data Fig. 2
Source Data Extended Data Fig. 3
Source Data Extended Data Fig. 5
Source Data Extended Data Fig. 6
Source Data Extended Data Fig. 7
Source Data Extended Data Fig. 9
