# Supplementary material for: Reducing brassinosteroid signalling enhances grain yield in semi-dwarf wheat
Source: Nature. 2023 Apr 26;617(7959):118–24. doi: 10.1038/s41586-023-06023-6 (PMC10156601; doi:10.1038/s41586-023-06023-6)
Supplement: Supplementary file 1 — Uncropped gel images. [file 41586_2023_6023_MOESM1_ESM.pptx]

## Slide 1
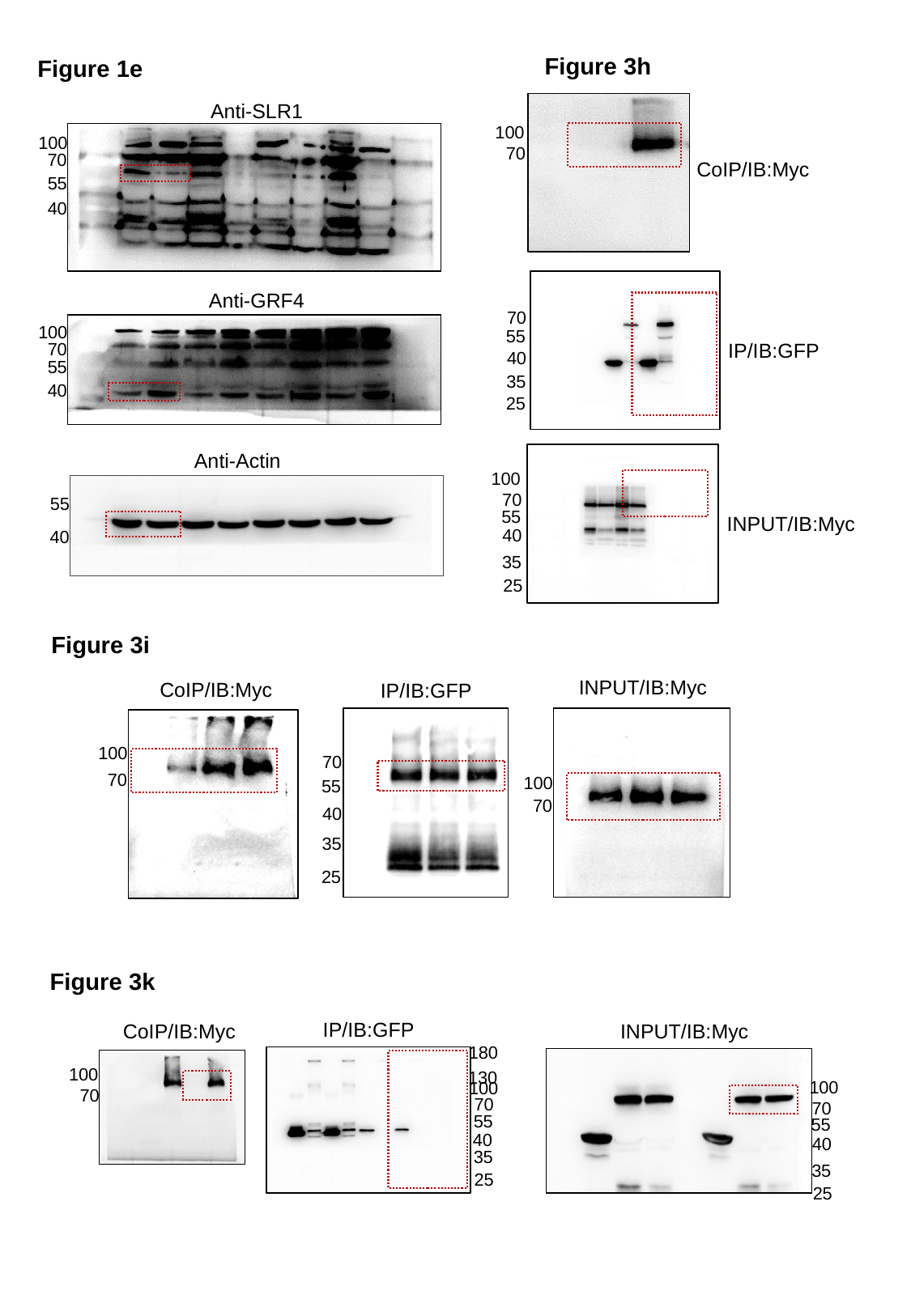

Figure 3h
Figure 1e
Anti-SLR1
100
70
55
40
100
70
CoIP/IB:Myc
70
55
IP/IB:GFP
40
35
25
Anti-GRF4
100
70
55
40
Anti-Actin
55
40
100
70
55
INPUT/IB:Myc
40
35
25
Figure 3i
INPUT/IB:Myc
CoIP/IB:Myc
IP/IB:GFP
100
70
70
100
55
70
40
35
25
Figure 3k
IP/IB:GFP
CoIP/IB:Myc
INPUT/IB:Myc
180
100
130
100
100
70
70
70
55
55
40
40
35
35
25
25

## Slide 2
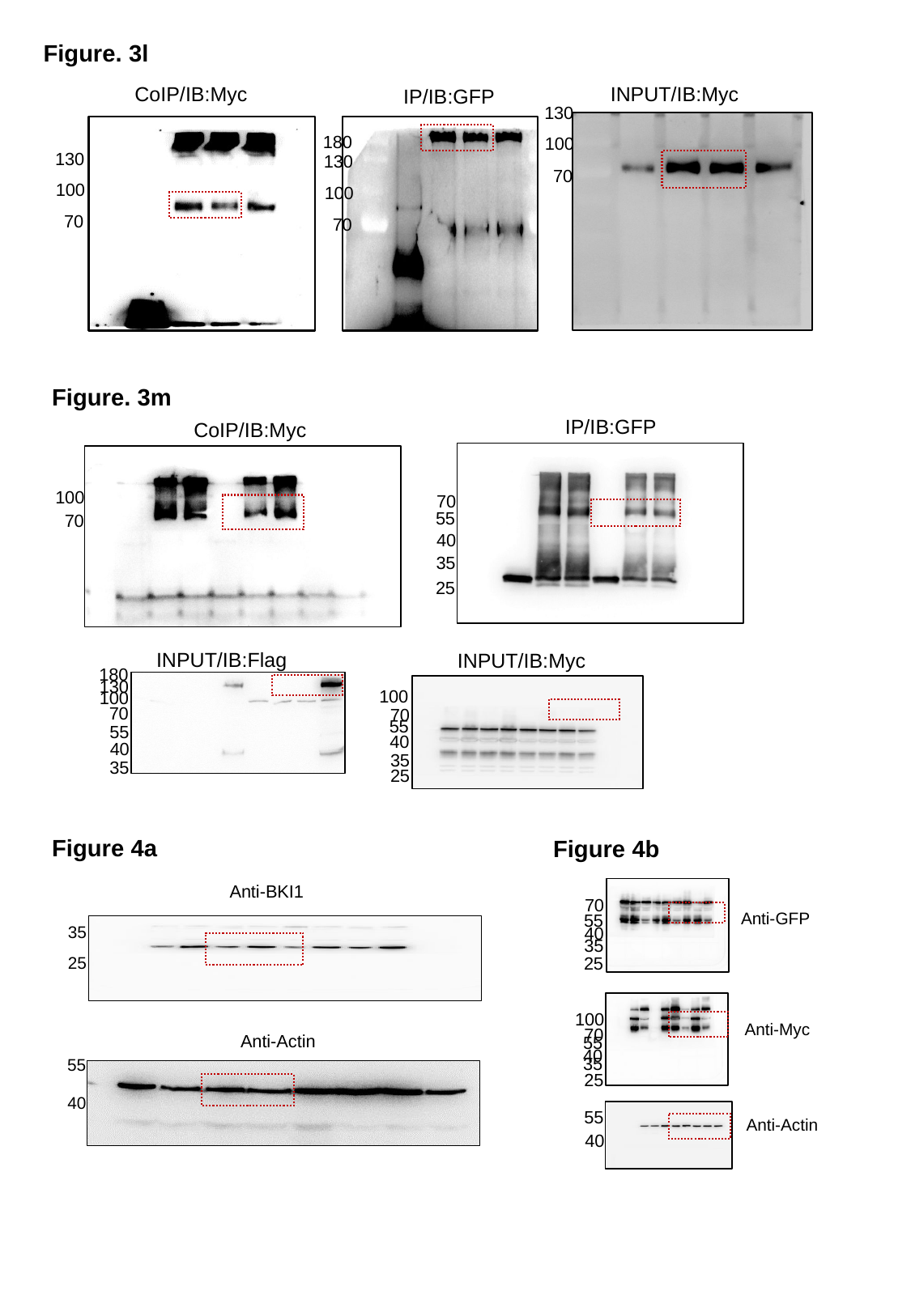

Figure. 3l
CoIP/IB:Myc
INPUT/IB:Myc
IP/IB:GFP
130
180
100
130
130
70
100
100
70
70
Figure. 3m
IP/IB:GFP
CoIP/IB:Myc
70
55
40
35
25
100
70
INPUT/IB:Flag
INPUT/IB:Myc
180
130
100
100
70
70
55
55
40
40
35
35
25
Figure 4a
Figure 4b
70
Anti-GFP
55
40
35
25
100
Anti-Myc
70
55
40
35
25
55
Anti-Actin
40
Anti-BKI1
35
25
Anti-Actin
55
40

## Slide 3
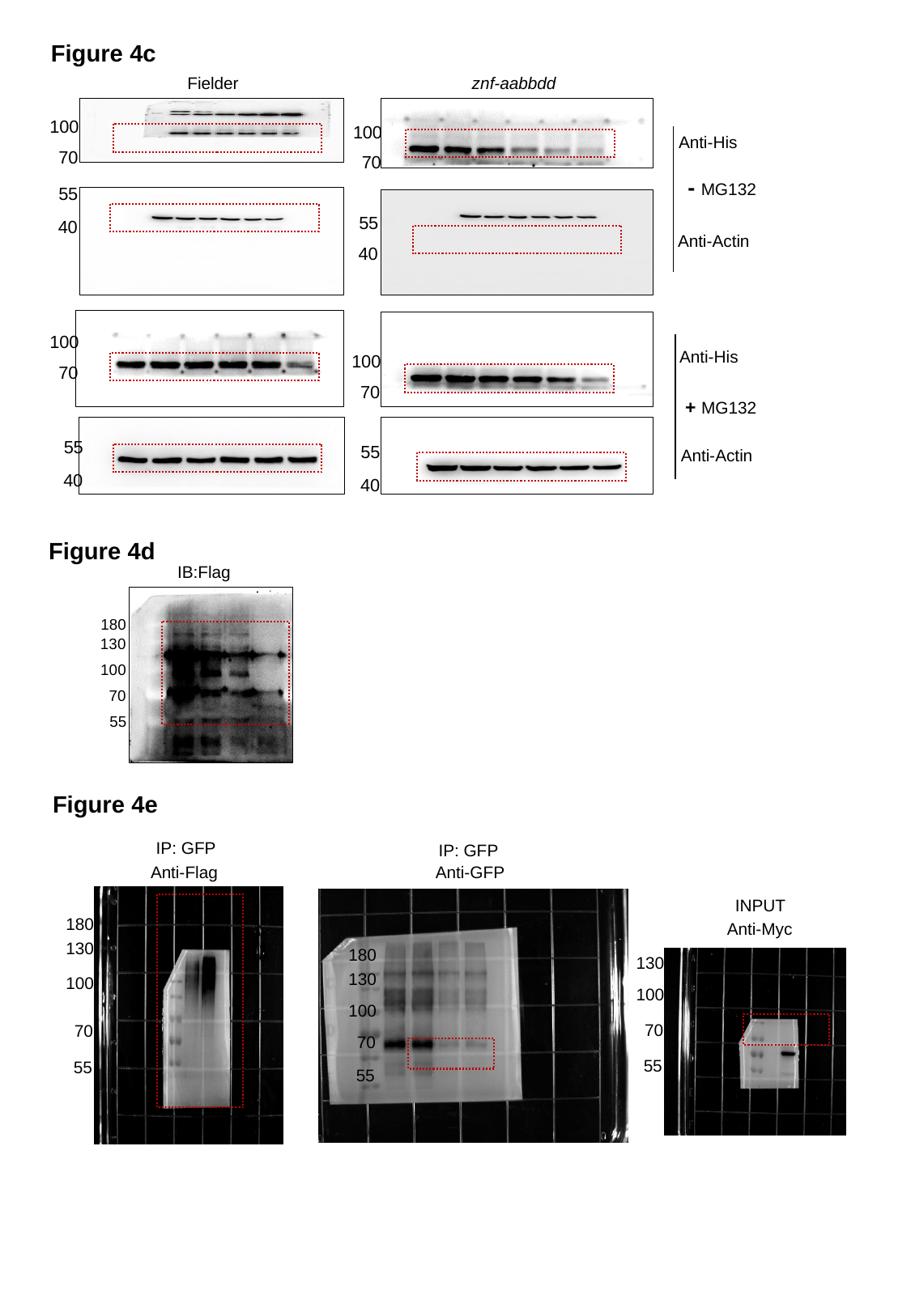

Figure 4c
Fielder
znf-aabbdd
100
100
Anti-His
70
70
- MG132
55
55
40
Anti-Actin
40
100
Anti-His
100
70
70
+ MG132
55
55
Anti-Actin
40
40
Figure 4d
IB:Flag
180
130
100
70
55
Figure 4e
IP: GFP
IP: GFP
Anti-Flag
Anti-GFP
INPUT
180
Anti-Myc
130
180
130
130
100
100
100
70
70
70
55
55
55

## Slide 4
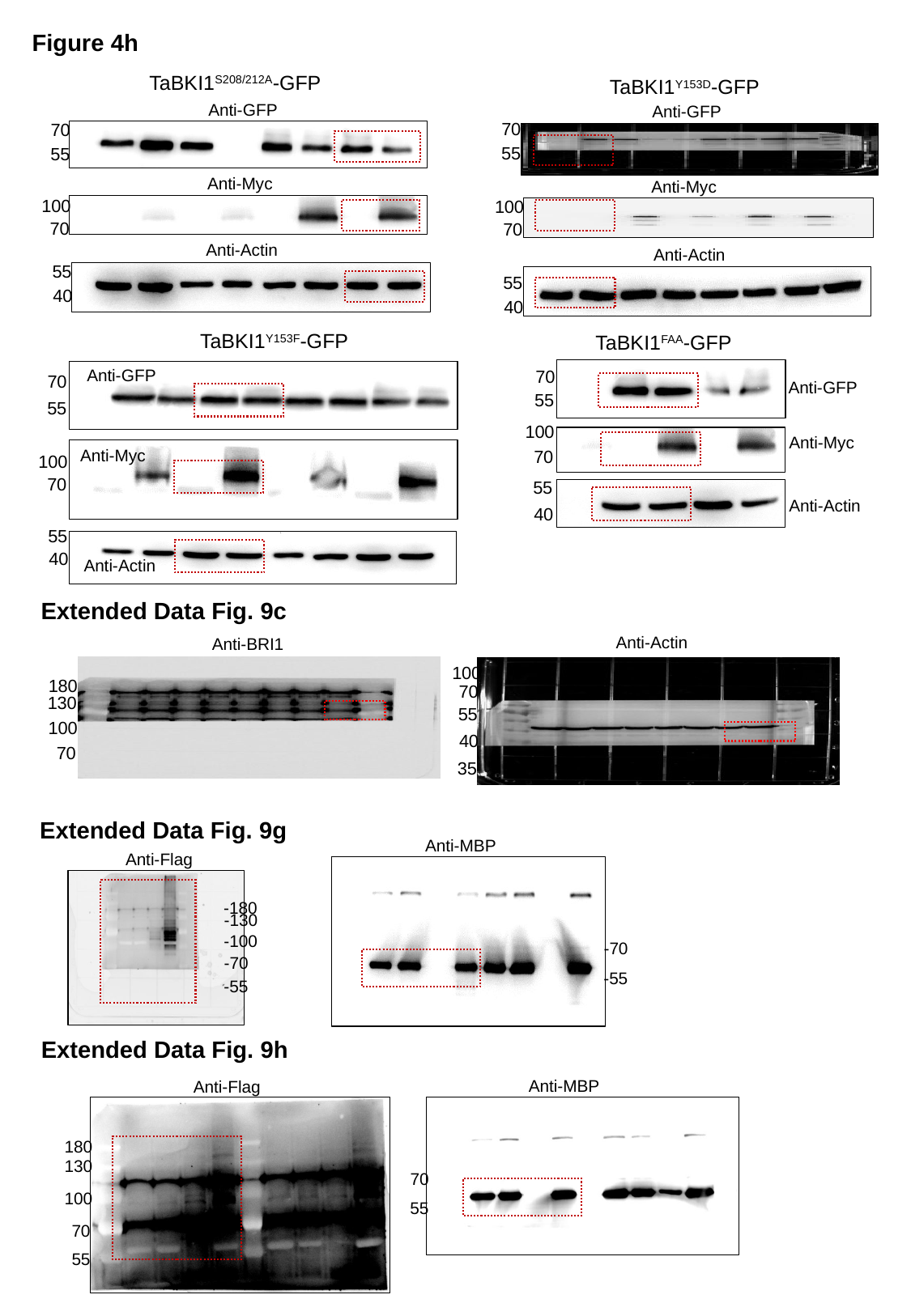

Figure 4h
TaBKI1S208/212A-GFP
TaBKI1Y153D-GFP
Anti-GFP
70
55
Anti-GFP
70
55
Anti-Myc
100
70
Anti-Myc
100
70
Anti-Actin
55
40
Anti-Actin
55
40
TaBKI1Y153F-GFP
TaBKI1FAA-GFP
Anti-GFP
70
α-ACTIN
Anti-GFP
55
100
Anti-Myc
70
55
Anti-Actin
40
70
55
Anti-Myc
100
70
55
40
Anti-Actin
Extended Data Fig. 9c
Anti-Actin
100
70
55
40
35
Anti-BRI1
180
130
100
70
Extended Data Fig. 9g
Anti-MBP
Anti-Flag
-180
-130
-100
-70
-70
-55
-55
Extended Data Fig. 9h
Anti-MBP
Anti-Flag
180
130
70
100
55
70
55
