# Supplementary material for: Reducing brassinosteroid signalling enhances grain yield in semi-dwarf wheat
Source: Nature. 2023 Apr 26;617(7959):118–24. doi: 10.1038/s41586-023-06023-6 (PMC10156601; doi:10.1038/s41586-023-06023-6)
Supplement: Supplementary file 2 — Reporting Summary [file 41586_2023_6023_MOESM2_ESM.pdf]

nature portfolio

Corresponding author(s): Zhongfu Ni
Last updated by author(s): Mar 5, 2023

Reporting Summary

Nature Portfolio wishes to improve the reproducibility of the work that we publish. This form provides structure for consistency and transparency in reporting. For further information on Nature Portfolio policies, see our Editorial Policies and the Editorial Policy Checklist.

Statistics

For all statistical analyses, confirm that the following items are present in the figure legend, table legend, main text, or Methods section.

n/a	Confirmed	
☐	☒	The exact sample size (n) for each experimental group/condition, given as a discrete number and unit of measurement
☐	☒	A statement on whether measurements were taken from distinct samples or whether the same sample was measured repeatedly
☐	☒	The statistical test(s) used AND whether they are one- or two-sided<br>*Only common tests should be described solely by name; describe more complex techniques in the Methods section.*
☒	☐	A description of all covariates tested
☒	☐	A description of any assumptions or corrections, such as tests of normality and adjustment for multiple comparisons
☐	☒	A full description of the statistical parameters including central tendency (e.g. means) or other basic estimates (e.g. regression coefficient) AND variation (e.g. standard deviation) or associated estimates of uncertainty (e.g. confidence intervals)
☐	☒	For null hypothesis testing, the test statistic (e.g. F, t, r) with confidence intervals, effect sizes, degrees of freedom and P value noted<br>*Give P values as exact values whenever suitable.*
☒	☐	For Bayesian analysis, information on the choice of priors and Markov chain Monte Carlo settings
☒	☐	For hierarchical and complex designs, identification of the appropriate level for tests and full reporting of outcomes
☒	☐	Estimates of effect sizes (e.g. Cohen's d, Pearson's r), indicating how they were calculated

Our web collection on statistics for biologists contains articles on many of the points above.

Software and code

Policy information about availability of computer code

Data collection	The fluorescence signal was detected using a confocal microscopy (LSM880, Zeiss).<br>Images from immuno blotting were collected with CLINX (ChemiScope 6000).<br>The LUC activity was analyzed using the Night SHADE LB985 (Berthold).<br>Illumina NovaSeq platform was used to collect the sequencing data.<br>Bio-Rad CFX96 with CFX Maestro 1.1 software was used to qPCR analysis.<br>The 15N content was measured using an isotope ratio mass spectrometer (Thermo Finnigan Delta Plus XP; Flash EA 1112).<br>For histological analysis, photographs were taken with a microscope imaging system (DS-U3, Nikon, Japan) and the cell lengths were measured with CaseViewer 2.3 (3DHISTECH, Ltd., Hungary).<br>For endogenous phytohormone quantification, GAs and BRs analysis was performed on a quadrupole linear ion trap hybrid mass spectrometer (QTRAP 6500, AB SCIEX).
Data analysis	Image analysis: ImageJ (version 1.45)<br>Statistical analysis: GraphPad Prism (version 7.00)<br>RNA-seq analysis: DESeq2 (v1.26.0)<br>Phylogenetic tree: MEGA5.0<br>Genetic diversity analysis: VCFtools (v0.1.13)<br>Single marker analyses: WinQTLCart (version 2.5)<br>Microcollinearity analysis:TriticeaeGeneTribe (Online tools: http://wheat.cau.edu.cn/TGT/; DOI: 10.1016/j.molp.2020.09.019)

For manuscripts utilizing custom algorithms or software that are central to the research but not yet described in published literature, software must be made available to editors and reviewers. We strongly encourage code deposition in a community repository (e.g. GitHub). See the Nature Portfolio guidelines for submitting code & software for further information.

Data

Policy information about availability of data

All manuscripts must include a data availability statement. This statement should provide the following information, where applicable:

- Accession codes, unique identifiers, or web links for publicly available datasets
- A description of any restrictions on data availability
- For clinical datasets or third party data, please ensure that the statement adheres to our policy

The authors declare that all data are available in the Article and Supplementary Information. All original gel blots are shown in Supplementary Fig. 1. Data points in graphs are shown in Source Data files. Raw data generated by this research have been deposited in the National Center for Biotechnology Information (NCBI) under accession number PRJNA852953 for RNA-seq.

Human research participants

Policy information about studies involving human research participants and Sex and Gender in Research.

Reporting on sex and gender	NA
Population characteristics	NA
Recruitment	NA
Ethics oversight	NA

Note that full information on the approval of the study protocol must also be provided in the manuscript.

Field-specific reporting

Please select the one below that is the best fit for your research. If you are not sure, read the appropriate sections before making your selection.

☒ Life sciences ☐ Behavioural & social sciences ☐ Ecological, evolutionary & environmental sciences

For a reference copy of the document with all sections, see nature.com/documents/nr-reporting-summary-flat.pdf

Life sciences study design

All studies must disclose on these points even when the disclosure is negative.

Sample size	No statistical methods were used to predetermine sample size. The sample size are described in the relevant figure legends and supplementary information, which enable researchers to conduct confident statistical analysis. Previous publications considered to determine sample size include: Agronomic traits(Liu et al., Nature 2020, 250: 600-605; Tian et al., Science 2019, 365: 658-664;); eBL or BRZ treatment(Zhao et.al., PNAS 2020, 117(35): 21766-21774)
Data exclusions	No data were excluded.
Replication	All experiments were repeated at least two or three times, and the number of independent experiments or biological replicates is indicated in the figure legends.
Randomization	All samples were collected randomly into experimental groups. The plant materials were grown under specific conditions and planting methods, which are described in detail in the methods.
Blinding	The research materials are plants so the blind design is not applicable in the field. For molecular biology experiments, bias could not be introduced since samples were treated identically and collected randomly. Experiments were repeated by different authors. The researchers also evaluated agronomic traits and performed RNA-seq analysis without prior knowledge of the results.

Reporting for specific materials, systems and methods

We require information from authors about some types of materials, experimental systems and methods used in many studies. Here, indicate whether each material, system or method listed is relevant to your study. If you are not sure if a list item applies to your research, read the appropriate section before selecting a response.

Materials & experimental systems

n/a	Involved in the study	
	☒	Antibodies
☒	☐	Eukaryotic cell lines
☒	☐	Palaeontology and archaeology
☒	☐	Animals and other organisms
☒	☐	Clinical data
☒	☐	Dual use research of concern

Methods

n/a	Involved in the study	
☒	☐	ChIP-seq
☒	☐	Flow cytometry
☒	☐	MRI-based neuroimaging

Antibodies

Antibodies used

Anti-GFP (Abcam, Cat# ab32146, 1:2000 dilution)
Anti-SLR1 (ABclonal, Cat# A16279, 1:1000 dilution)
Anti-GRF4 (ABclonal, Cat# A20348, 1:1000 dilution)
Anti-MYC (California Bioscience, CB100002M, 1:2000 dilution)
Anti-BRI1 (Setaria italica, SiBRI1, 1:1000 dilution)
Anti-BKI1 (custom-developed by ABclonal® Technology, China, 1:1000)
Anti-Flag (Sigma, Cat# F1804, 1:2000 dilution)
Anti-His (EASYBIO, Cat# BE2017, 1:2000 dilution)

Validation

Validation statements and experiments can be obtained from the following websites and publications:
Anti-GFP (https://www.abcam.cn/gfp-antibody-e385-ab32146.html)
Anti-SLR1 (https://abclonal.com.cn/catalog/A21231)
Anti-GRF4 (https://abclonal.com.cn/catalog/A20348)
Anti-MYC (http://www.seajetsci.cn/docs/cali-bio/CB100002M.pdf)
Anti-BRI1 (DOI: 10.1073/pnas.2002278117)
Anti-BKI1 (custom-developed by ABclonal® Technology, China, 1:1000)
Anti-Flag (https://www.sigmaaldrich.cn/CN/zh/product/sigma/f1804)
Anti-His (http://www.bioeasytech.com/product/2386.html?goods_id=4371)
